# Unicanvas: Exploring a tool for strategic management

**DOI:** 10.12688/openreseurope.17233.1

**Published:** 2024-04-11

**Authors:** Julio Segundo, Mariluz Fernandez-Alles, Maria Velez, Jose M. Sanchez

**Affiliations:** 1 Business Organization Department, University of Cadiz, Cádiz, Andalusia, Spain; 2 Department of Financial Economics and Accounting, University of Cadiz, Cádiz, Andalusia, Spain

**Keywords:** Strategic management, University, Business model canvas, Higher Education Institutions

## Abstract

**Purpose:**

This research proposes an adapted version of Business Model Canvas (BMC) as a strategic instrument for Higher Education Institutions (HEIs). University-Model Canvas (Unicanvas) is intended to be a critical visual and dynamic tool for addressing the challenges faced by entrepreneurial universities in their quintuple helix model.

**Research methods/Approach:**

Considering the new challenges facing HEIs, transfer knowledge, corporate social responsibility, internationalization, digitalization, sustainability, and the critical role of universities in innovation and regional development, we adapt the strategic building blocks of the BMC in the context of universities.

**Findings and implications:**

Departing from BMC, we propose 10 strategic building blocks against the nine BMCs, including an achievement box. The new challenges HEIs face, the expansion of university outputs, and new societal demands highlight the opportunity to use this tool instead of traditional and static strategic planning models to discuss and concretise their differentiated way of competing. The use of this participative, dynamic, intuitive, and flexible strategic tool will facilitate the holistic strategic management of these institutions in their current new challenges.

## Plain summary

Universities are responsible for training students, generating knowledge from their research, and transferring it to the market. This triple function is developed in a changing context with significant challenges, such as demographic changes, rapid technological advances, or limited public financing. Changes for Higher Education Institutions (HEIs) imply contemplating key issues such as transfer and innovation, internationalization, digitalization, sustainability, or regional development in their strategic objectives. For this reason, many universities are asking themselves: How can they approach the strategic management of universities to face these challenges? Universities have traditionally approached the management of their institutions based on traditional and static strategic planning models. However, universities with more attractive strategic tools from the business and entrepreneurship fields could design strategies to meet the interests of multiple academic and market stakeholders. The original Business Model Canvas (BMC) is a widely accepted tool for business model innovation. It provides an easy, dynamic, intuitive, and flexible vision that can be continually improved to shape and renew any business strategy. The main goal of this research is to propose a modified version of BMC, named University-Model Canvas (Unicanvas). A Unicanvas is proposed for its effectiveness, practicality, and easy-to-use tool design and development of university strategic management. We aim to help university managers and administrators understand the possibilities of this tool for appropriate strategic management in these changing times.

## Introduction

Higher Education Institutions (HEIs) have adopted different roles as a result of the transition from the triple helix model to the entrepreneurial university, where in addition to teaching, research, and transfer, universities are participants in economic development, taking into consideration social and natural context ( Carayannis & Campbell, 2011; European Commission, 2020; Garcia Aracil, 2013; Garcia *et al.*, 2022). The engaged university is currently emerging, where it is also sensitive to regional issues ( Thomas *et al.*, 2023). In this context, universities are responsible not only for the qualification of students and research and knowledge creation but also for responding to the demands of the market and the needs of society and engaging their activities in social and economic contexts ( European Commission, 2020; Thomas *et al.*, 2023). Furthermore, universities face external and internal challenges, which include demographic student changes, increasing higher education demands, increasing competition, international and new emerging models of higher education, society contingencies, increased cost education, lower government funding, rapid technological advances, digitalization, and new entrepreneurial/ethics/sustainable/social standards ( Garcia *et al.*, 2022; Guilbault, 2016). As a result, many universities have shown interest in their strategic management process ( Garcia Aracil, 2013; Vlachopoulos, 2021). The dual economic and social mission of universities, the multiplicity and complexity of different roles of universities, and the challenges they must face have made the strategic management of HEIs complex ( Thomas *et al.*, 2023).

Over the decades, universities have carried out their strategic management following traditional and static strategic planning models. In this sense, most HEIs have shown a normative isomorphism, developing strategic plans according to the same scheme. However, these models have certain limitations. Firstly, strategic plans tend to work with a four-year time horizon and are updated after this period, which implies inertia and little flexibility and dynamism. Secondly, these strategic plans have been designed by top management, resulting in plans with little participation, which means that implementation is sometimes not very well supported. Thirdly, while many challenges are shared among universities, each institution must specify its way of addressing them, *i.e.* what kind of university it wants to be, what its value is, and what its strategic model will be, questions that are difficult to answer with an isomorphic strategic formulation. For these reasons, static models need to adequately address the new circumstances of HEIs marked by the complexity of their roles and the social and economic challenges. In consequence, new strategic tools are called to define dynamic models ( Garcia Aracil, 2013; Vlachopoulos, 2021).

The development of iterative approaches such as design thinking and joint inquiry techniques in HEIs that allow management practitioners to jointly inquire into specific strategic management problems has led to the use of visual tools such as business model canvas (BMC) of Osterwalder and Pigneur (2010) ( Avdiji *et al.*, 2020). BMC is a broadly accepted tool for supporting business model innovation. In a single-page view, BMC provides an easy, visual, dynamic, intuitive, and flexible tool that can be developed and continuously improved to shape and renew any business strategy. BMC can also highlight the alignment of top strategies and bottom actions, which enhance strategic competitiveness and even discover previously unseen opportunities for value creation. For all these reasons, BMC has been used in different sectors, for other types of companies, and to explain different performances. Existing adaptations of these tools exist, such as lean canvas, ecocanvas, sustainable business canvas, or value propositions canvas.

In the context of HEIs, despite being a tool that is taught for entrepreneurship courses in the leading universities ( Türko, 2016), BMC has been timidly used, but either in a partial way or to achieve some specific strategic objectives ( Dahlan *et al.*, 2020; Gaus & Raith, 2013; Gomes *et al.*, 2020; Hadăr *et al.*, 2016; Jongbloed & Kottmann, 2022; MacBryde & Franco-Santos, 2016; Mulyana *et al.*, 2018; Polat, 2022; Rytkonën & Nenonen, 2014; Trevisan *et al.*, 2022; Trunina *et al.*, 2021).

This research aims to propose a modified version of BMC, named University-Model Canvas (Unicanvas). A Unicanvas is suggested for its effectiveness, practicality, and easy-to-use tool design. This proposal is not just an illustration but a discussion of the implications of the challenges faced by HEIs in the design of their strategic models. The main contribution of the document is to provide a dynamic and visual tool particularly suitable for defining HEI strategic models.

The paper is structured as follows. After this introduction, the importance of strategic management for HEIs is discussed. Subsequently, an analysis of the origins and elements of Unicanvas is discussed. Finally, the conclusion, discussion, contributions, implications, and future lines of research are presented.

## Strategic management in Higher Education Institutions

Traditional models such as Porter's Five Forces, BCG growth-share matrix, and Strategic group maps carry out the management process analytically and rationally based on deductive and inductive processes leading to organization and planning. However, "moving beyond traditional strategic management approaches, joint inquiry through design thinking techniques has emerged as a valuable approach in strategy making" ( Avdiji *et al.*, 2020: 696). Unlike traditional models, visual inquiry tools develop a joint inquiry process where alternative hypotheses are explored. These tools have a creative and iterative design based on abduction and invention and in design thinking techniques. They work well with ambiguity and uncertainty, are mainly visual, and are based on sketching and prototyping, intensive observation and wondering, and challenging stereotypical perception ( Avdiji *et al.*, 2020). One of the first and best-known visual inquiry tools that have transformed business modelling by providing a design space framed by nine building blocks is BMC.

BMC of Osterwalder and Pigneur (2010) is a holistic, visual, simple, dynamic, participative, unbureaucratic, intuitively understandable, and practical tool for the design of the logic of a firm. Nowadays, the BMC is the most commonly applied tool for business model design ( MacBryde & Franco-Santos, 2016). It takes up the nine strategic building blocks for the value creation process transformation. It assigns them to the four most important areas of the company: offer, customers, infrastructure, and financial structure. The nine blocks are Customer Segments, Value Propositions, Customer Channels, Customer Relationships, Revenue Streams, Key Activities, Key Resources, Key Partners, and Cost Structure.

Although there are precedents for using BMC in the context of HEIs, mainly it constitutes a tool that is taught for entrepreneurship courses in leading universities ( Türko, 2016), it has been timidly used for the strategic management of universities, but either in a partial way or to achieve some specific strategic objectives. For example, Gaus and Raith (2013) developed a general BMC for entrepreneurial universities. Rytkonën and Nenonen (2014) strove to ascertain how BMC could contribute to university campus management. MacBryde and Franco-Santos (2016) defend the use of BMC for university strategic management by providing a tool for effective strategic management that considers the complexity of universities. Specifically, they proposed that departments and centres should be given free rein to design their own "sustainability model" by developing a more appropriate performance measurement system. Hadăr *et al.* (2016) used it for value propositions and growth strategy in the case of technology transfer centres for valorising intellectual property rights. Others, such as Gomes *et al.* (2020), applied BMC in the context of the digital transformation of HEIs. Trunina *et al.* (2021) also developed BMC in a university context, but only for study programs. Jongbloed and Kottmann (2022) have applied to BMC for universities, but only for value proposition analysis. Trevisan *et al.* (2022) used BMC for HEI eco-innovative strategies, Polat (2022) applied BMC in techno parks analysis. Finally, Mulyana *et al.* (2018) and Dahlan *et al.* (2020) have combined in the university context BMC with other strategic tools such as SWAT, Environmental Map, and Value Proposition Design Canvas.

The marketization of HEIs highlights the opportunity of using BMC as a strategic tool that follows the principle of value creation. The application of the BMC in the context of HEIs as a university strategic tool could enable a holistic, agile, and visual picture of their strategic processes to be generated. Also, BMC allows changes or adjustments, which occur very often in a university context, to be implemented easily and quickly. Finally, BMC is a tool characterised by a high degree of participation. With these antecedents, this article proposes Unicanvas as a practical and dynamic tool to address their strategic concerns from a unique holistic point of view.

## Building Unicanvas

### Origins

The definition of a business model is adopted herein, as stated by Osterwalder and Pigneur (2010), according to which it describes the rationale of how an organization creates, delivers, and captures value. BMC is a conceptual tool that many scholars and practitioners have widely adopted. BMC involves translating strategic management concerns, such as positioning and goals, into a visual model that deploys how businesses function. This framework enables the business structure and systems to be designed by conceptualizing an organization through three key aspects: how key components and functions are integrated to deliver value, how they are interrelated within the organization and across its supply chain and stakeholder networks, and how the organization generates value, or creates profit, through those interconnections. This visual tool classifies businesses' processes and internal activities into nine strategic and interlinked categories, each representing a building block.

1. The first block symbolizes the heart of any business model, which is that of customer segments.
2. The value proposition includes combining products and services that create value for customer segments.
3. Customer relationships explain the relationship the business wishes to develop with every customer group.
4. Channels describe the paths a company employs to reach its target customers in delivering its desired value proposition.
5. Revenue streams represent the income a company is reaching.
6. The sixth strategic block, key resources, reveals the most critical assets needed.
7. Key activities are the main tasks and actions that should be deployed.
8. The eighth block identifies the key partners in terms of stakeholders and associates.
9. Cost structure gathers the most critical operational expenses and costs.

Although presented sequentially, the definition of box contents for each business is an iterative process. Each box is interrelated, meaning that defining the contents of one box affects the contents of others. This, in turn, may require redefining the contents of the preceding ones. The interrelationships of the boxes give it a dynamic character and make it a tool that allows for rapid changes.

## Unicanvas

**Figure 1.**
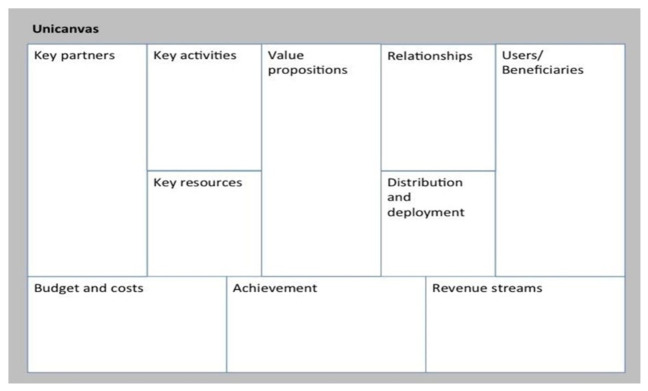
Unicanvas.

The different elements included in the Unicanvas tool are shown in Figure 1.

Following the canvas philosophy, the process of fulfilling in the boxes is an iterative process, usually made with post-its where each one represents one idea defined with three or four words. The reflection and decisions taken in one section condition the next one, but at the same time, the reflection on the following boxes can lead to a reconsideration of the initial decision. In the current context of HEIs, this tool also allows to establish potential scenarios when there are uncertainties associated with the process and to reflect on changes to be undertaken in a stratified manner. Even more, a university could develop alternative routes or models to reach the agreed vision, several different canvases, to analyse different possible strategies, looking at their implications, or for different beneficiary segments. Because the proposed blocks are easy to understand, with Unicanvas, the planning process is participatory, and the potential benefits can be integrated into the process. Taking into account these considerations, the process should be developed in the following stages.

First, the key beneficiary identification process precedes the other boxes and is the key to the whole process. This building block answers the question: For whom is value created? ( Osterwalder & Pigneur, 2010). HEIs' marketization implies that universities need to become more market-oriented and include a customer orientation in their strategic planning ( Bunce *et al.*, 2017; Guilbault, 2016). One of the significant problems, however, involves the identification of the university customers. Traditionally, students were considered the customers of universities. Despite its popularity, the assumption that the student is the customer remains unclear and open to debate ( Mark, 2013). According to Gauss and Raith (2013), it is mainly in private universities where students are viewed as customers and are offered expert knowledge and a job-qualifying degree as a means of value. The government has defined students as customers in England by introducing student tuition fees ( Bunce *et al.*, 2017). Even in particular universities, students have a customer's perception and claim the rights observed in the everyday marketplace. In this line, Mark (2013) concludes that universities must continue adopting the student-customer paradigm to increase the quality of their instruction, the accessibility to the faculty, and the improvement of the efficiency of their processes.

However, students are just one customer segment. HEIs, like other industries, have multiple customers and beneficiaries ( Guilbault, 2016). In this respect, Gauss and Raith (2013) develop different BMC by considering different segments such as students, firms, start-ups, research institutions, the government (as an employer), the research community, innovative firms, R&D units, and innovative customers. The tool itself makes it possible to differentiate each of the beneficiaries based on identifying colours or even making different canvases for each beneficiary. Thus, given that the needs and concerns of these potential customers may diverge significantly, this building block becomes complex and problematic.

As an illustration of these ideas, and by considering diffuse differences between customer and beneficiary in this context, each university should use this category to define its beneficiaries broadly. Nowadays, the value created by universities is much broader, and the impact of universities on innovation and regional development means that the number of users and beneficiaries is ever-increasing. In this regard, in terms of the four missions of universities, teaching, research, transfer, and social commitment, we can identify the following beneficiaries. Students, alumni, and lecturers, in the case of teaching; researchers and academicians, for research; spin-offs and start-ups, external firms, employers, and the government and its administrators, for transfer; and families, and society for the last of its functions. From this wide variety of beneficiaries common to all universities, we propose that in this box each university should select only the key beneficiaries to develop the desired future of the institution. Those that will be the focus of its strategy, i.e., different universities could give greater emphasis to one of their missions, and therefore focus more on one type of beneficiary or even different universities may identify the same key beneficiaries, but there may be differences in the specific segment of that beneficiary on which they focus. For example, certain universities located in older populations may focus their strategy on attracting foreign students and these may be their key or try to find more students in the lifelong learning niche. The choice made will condition the decisions and choices of the other boxes.

Second, once the beneficiaries have been identified the next process is the value propositions, precisely targeted at those key beneficiaries. This category answers the questions: What value is offered? What problems are solved? Which needs are fulfilled? Which goods or services are provided? ( Osterwalder & Pigneur, 2010). The core of BMC involves the proposition of the firm's value, which defines the kind of value supplied. In the context of the quintuple helix, HEIs provide complex offers since they arise from teaching, research, and technology transfer and in response to the society and natural environment.

Hence, to make a difference, we propose that each university designs its value propositions as a unique combination of products and services that provide differential value to its key various beneficiaries. Thus, for the traditional consumer, students, valuable education and training opportunities, proximity, location, and physical structures. The traditional aim of universities is teaching, that is, to disseminate knowledge to students to shape them into first-class graduates who will be hired by public or private firms ( Gauss & Raith, 2013). Likewise, for researchers and academicians, the proposition of the value of an HEI as a research institution is evaluated by its research infrastructure incentives but also by quality indicators ( *e.g.*, number of citations, research projects, and contracts, and awards), range from publications to patents that document the outcomes of their innovative research. In the third mission, for spin-offs, start-ups, and external firms, universities as R&D transfer, institutions offer marketable products, services, technologies ( *e.g.*, number or revenue from patents, licenses, contracts, and academic spin-offs), and clustering, incubation, acceleration programs, networking opportunities, and Technology Transfer Office (TTO) services. Similarly, universities currently go beyond these three broad activities to assume a crucial and central role in regional development, key for the government and society, by engaging in new activities to offer economic, social, and cultural contributions and legitimization and recognition. Starting from the selected key beneficiaries, the university will discuss and specify which needs will be covered.

Third, beneficiary relationships. This category answers the question: What is the relationship with each customer segment? ( Osterwalder & Pigneur, 2010). As we have proposed, identifying key beneficiary segments is complex for HEIs. Therefore, the complexity of strategic management is accentuated when different segments require different relationships because one of the universities' main goals is to develop longstanding value from reciprocally valuable relationships with each beneficiary segment. According to Gauss and Raith (2013), universities could strengthen their relationships through mentoring programs, student organizations, internships, TTOs, business incubators, academic conferences, R+D networks, and student programs.

Therefore, each university can design its routines/protocols and describe how it establishes, maintains, and enhances contact with beneficiary segments, i.e., it should establish activities to attract and maintain beneficiaries, *e.g.*, students, or prestigious researchers. For students, personal assistance and orientation through assisting, guiding, and mentoring programs. For researchers and academicians, advising and informing programs and participating in and valuing co-creation are essential. And for other beneficiaries by creating and maintaining communities or ecosystems around HEI. Consequently, strategic communication units are key in establishing and maintaining a good relationship with key beneficiaries and promoting the programs and services provided, *e.g.*, websites, social media, mailing, or fairs and exhibitions. 

Forth, how distribution and deployment channels are established. This block answers the question: Which are the main distribution channels? ( Osterwalder & Pigneur, 2010). Value propositions of universities are delivered through different distribution channels to their beneficiaries ( MacBryde & Franco-Santos, 2016). According to Gauss and Raith (2013), universities must develop job platforms, courses, seminars, journals, publishers, calls for proposals, start-ups, academic spin-offs, and transfer units.

In 2016, Steve Blank created Mission Model Canvas, an alternative to the BMC for military units. This variant proposes relabelling this category as deployment instead of simply distribution. Following his arguments, in the business world, we should ask: What type of distribution channel do we choose to deliver the product/service from our company to the customer segments? However, for the US Department of Defense, Blank (2016) proposes: How can we deploy the product/service for widespread use among people who need it? What channels can we use, and how can we communicate our value proposition to our beneficiaries? In this direction, it is necessary to define some key aspects, such as establishing the message distribution channels, outlining a promotion plan, or identifying the customer segment involved in the action.

For universities, this box implies getting in touch with its beneficiaries, delivering its value proposition, and creating the beneficiary experience ( Osterwalder & Pigneur, 2010), *e.g.*, through educational programs and events organized by or involving HEIs, such as training courses, seminars, conferences, meetings, competitions, company congresses, and networking events. Therefore, choosing appropriate channels for each beneficiary segment and the effective use of channels delivers, and raises awareness of the services provided. Accordingly, each university should design its distribution and deployment channels and describe the service and communication distribution channels required to present its value to its beneficiaries. Intranet, e-learning, instant messaging, voice and video conferencing tools, meetings, seminars, conferences and congress, hackathons, document collaboration tools, task management tools, or networking events should be designed. Not all universities should give equal importance to all channels. Depending on the key beneficiaries chosen, some channels will be more strategic than others.

The fifth category, revenue streams, answers the question: Which values are being paid for? How are payments made? What are the relative shares of individual revenue streams? ( Osterwalder & Pigneur, 2010). Private and public universities need to indicate ways to obtain financial flows and sustainability of their model. Furthermore, this category describes the available funding sources, the budget, and the financial configuration required to deliver the value proposition.

The growing cost of the system has forced developed countries to change their university incomes, which were initially dependent only on public financial support. Public universities are also encouraged to find new sources of revenue through activities that place them in the knowledge-based economy. As Gaus and Raith (2013) argue, each university should indicate various sources of income, with different percentage weights, not only in the form of student fees, research grants, and public government funds but also as transfer income, such as equity stakes, patent sales, licensing and contract income, service income and product sales. In addition, each university must establish its revenue streams from rental fees (conference rooms or land), property management, and service fees. For example, Mulyana *et al.* (2018) identified a business model development strategy for Padjadjaran University, to alter their general university management to be more creative in finding other revenue streams to maintain their sustainability.

In sixth place, key activities should be defined. This category answers the question: Which activities for value creation are required? ( Osterwalder & Pigneur, 2010). According to Gaus and Raith (2013), universities could develop key activities, such as teaching, research, transfer, proactive market participation, and product development. Recently, university challenges have contributed to the deployment of new activities, such as social responsibility, sustainability, entrepreneurship, and regional development.

Considering the contents of prior building blocks, each university must describe how its key activities are organized by providing the initiatives required to generate value for its beneficiaries/users. Consequently, key activities such as administration, property management, facilities management, corporate communication, marketing and networking, entrepreneur support, technologies assisting, incubation programs, industry-university cooperation, IPR licensing, and so on should be considered. Remember that, similarly, the selection carried out in the previous boxes determines the key activities. For example, a university that has chosen to internationalise its students through agreements with Asian universities should prioritise this establishment of agreements, or one that chooses to become a lifelong university may prioritise e-learning. 

Seven, key resources. Likewise, this box answers the question: Which resources are required for value creation? ( Osterwalder & Pigneur, 2010). Various key resources for value creation, such as teaching staff, students as input, senior and junior scientists, a complete research infrastructure, research findings, researchers, and transfer experts, can be found. These resources are supported by the university infrastructure and administration, technological support, science parks, academic and business networks, university incubators, and efficient TTOs ( Gaus & Raith, 2013).

We also suggest that universities describe their critical resources, whether physical or intangible, to offer value to their beneficiaries. Human resources, talent, knowledge, technological resources (laboratories or equipment), financial resources, and credibility are all essential for universities. But also, key properties, financial resources, management competencies, and even image and reputation.

Eight, key partnerships. This category answers the question: Who are the most important partners for value creation? ( Osterwalder & Pigneur, 2010). Value creation also requires the support of a network of associates to produce and deliver this value. According to Gaus and Raith (2013), partners could include not only teaching staff, firms, public institutions, and researchers from other universities or research centres but also business incubators, scientific parks, venture capitalists, business angels, and, of course, alumni. In this regard, universities require the establishment of long-term and closer relationships with partners from different contexts.

HEIs must describe their network of cooperation agreements that seek to deliver higher values and should include other potential partnerships. Unicanvas could help by identifying current partnerships and disclosing potential external clients for future contacts. Other universities, research institutions, government institutions, public and private agencies, local government, cluster, and organized industrial areas, chambers of commerce, business associations, and technoparks should be considered. For example, if we have chosen dual programmes as a key strategy, one of our key partnerships should be companies and institutions that offer work experiences and internships.

Nine, budget and cost structure. This category answers the questions: Which are the most important expenditures? Which activities/resources create the highest costs? ( Osterwalder & Pigneur, 2010). The strategic plan includes the final costs derived from its structure and means involved in the operation of the model. In this respect, Gaus and Raith (2013) describe the personnel expenses (such as salaries of academic and administrative personnel) and the infrastructure and administration costs. Furthermore, in addition to financial costs, ecological and social costs of universities should be included.

Similarly, the mission model canvas proposes that organizational budgets should match the cost structure. In this respect, each solution needs to be evaluated based on the budget of the required solution ( Blank, 2016). To depict these arguments, universities need to identify the main costs associated with this model, including financial, environmental, and social impacts, as well as their budget. Consequently, each university could consider initial, maintenance, and operational costs.

Finally, achievement, fulfilment, and impact. To the strategic building blocks of BMC, we propose to add a block in the Unicanvas. Following the mission model canvas ( Blank, 2016), the US Department of Defense mobilized resources and budgets and created value for a set of beneficiaries. Blank (2016) argued that the revenue streams box makes no sense in this setting and relabelled the previous block as mission achievement/success. Blank defines achievement as the value created for the sum of all of the beneficiaries/the greater good, which can be measured in a variety of ways, like the number of refugees housed and fed, the number of soldiers saved from roadside bombs, or the number of cyber-attacks prevented. None of these is measured in monetary terms, but all deliver value to the end beneficiary.

In the university context, the term 'business' or 'revenue streams' can be questionable as the overall mission of the majority of universities does not involve making a profit, as argued by MacBryde and Franco-Santos (2016). Similarly, Gaus and Raith (2013) also accentuate various university non-monetary revenues, such as university reputation, journal publications/editorships, and the number of patents. We defend that the previous category can be complemented with an added building block to depict these arguments. Therefore, this new category can be more suitable for collecting data for strategic planning at the university. Hence, in the Unicanvas model, we propose that each university could have its own 'achievement box'.

## Conclusions

HEIs are facing significant challenges that require their business models to be designed with more agile, visual practices, intuitive and dynamic, and less bureaucratic tools. Traditional models of university strategic management, criticized for their static and isomorphic nature, need to evolve towards models focused on value creation, such as BMC.

The paper's main contribution is that Unicanvas provides a starting point for the consideration, discussion, and transformation of the logic of strategic management in HEIs and how it should be delivered. Unicanvas could facilitate the distribution of strategic management knowledge (and its key drivers), enhance the understanding of competitive variables, and improve activity choices, resource allocation, and decision-making.

Encouraging the participation of the different actors involved in the process, Unicanvas is a tool that facilitates the integration of the debate of the different stakeholders involved in the reflection. Strategic planning is normally considered the main element, but not the essence, of strategic management, which instead uses several phases considered outside of the realm of planning (in the strictest sense of the term) that are related instead to resource management, the implementation of activities and processes, and control and evaluation. Unicanvas facilitates the definition of key performance indicators, as it facilitates their evolution to the Balanced Score Card ( Sánchez *et al.*, 2016). Furthermore, Unicanvas simplifies the implementation of the strategy, defining key activities, resources, allies, and costs, addressing one of the main problems for strategic management, especially in universities, where strategic plans are often established for four or five years that are neither developed nor followed up. 

Some theoretical implications can be derived from our proposal. First, Unicanvas, specifically the identification of the beneficiaries’ segment, could enrich the focus of the Stakeholders Theory by better identifying beneficiaries through the process of mutual value exchange beyond the process of resource transaction ( Falqueto *et al.*, 2020; Freudenreich *et al.*, 2020; Polat, 2022). Second, blocks of key partners, resources, and activities could be underpinned by the Resource-based View. This theoretical approach would help to classify the main value-generating resources of HEIs. Third, Network Theory could shed light on identifying the relationship block. Finally, Institutional Theory should be taken into account in the design of Unicanvas, assuming that universities from the same institutional context will share some blocks.

This research has practical implications, encouraging administrators to develop visual thinking for the business model of HEIs. Those responsible for university management should think in terms of value creation and reflect in a particular way on each of the boxes of the Unicanvas, assuming that their institutions' roles are increasingly diverse, and the complexity of management is increasing. Also, Unicanvas would have some implications in the HEI context for policymakers by allowing them to influence the design of an institutional framework to improve the functioning of universities and their value creation.

Future lines of research should be oriented, on the one hand, towards applying Unicanvas to the specific case of a university or consortium of universities. On the other hand, to propose complementary tools to Unicanvas to solve the deficiencies already pointed out in the BMC literature.

## Ethics and consent

Ethical approval and consent were not required.

## Data Availability

No data are associated with this article.
