# Unicanvas: Exploring a tool for strategic management

**DOI:** 10.12688/openreseurope.17233.3

**Published:** 2024-12-05

**Authors:** Julio Segundo, Mariluz Fernandez-Alles, Maria Velez, Jose M. Sanchez

**Affiliations:** 1 Business Organization Department, University of Cadiz, Cádiz, Andalusia, Spain; 2 Department of Financial Economics and Accounting, University of Cadiz, Cádiz, Andalusia, Spain

**Keywords:** Strategic management, University, Business model canvas, Higher Education Institutions

## Abstract

**Purpose:**

This research proposes an adapted version of Business Model Canvas (BMC) as a potential strategic tool for the design of the business model of Higher Education Institutions (HEIs). In the context of fifth-generation universities, the University-Model Canvas (Unicanvas) offers a solution to some limitations of traditional strategic tools. Unicanvas aims to be a critical visual and dynamic tool to address the new challenges faced by universities such as internationalization, digitalization, sustainability, and corporate social responsibility.

**Research methods/Approach:**

We adapt the strategic building blocks, in which some of the BMC blocks have been renamed and an achievement block has been added. We reflect theoretically on the peculiarities of each block in the context of universities to explain the versatility of the tool for designing university business models tailored to different value propositions and for various beneficiaries.

**Findings and implications:**

Unicanvas offers universities the flexibility and dynamism to adapt their different business models to various forms of value creation that arise from their growing number of beneficiaries, missions, and the new trends and challenges they face. We conclude that using this participatory, dynamic, intuitive, and flexible strategic tool will facilitate the holistic design of the business model of these institutions.

## Introduction

Higher Education Institutions (HEIs) have traditionally assumed the triple mission of teaching, research, and transfer. Nowadays, the role of universities is also highlighted in the model of the quintuple helix as participants in social and economic development, considering natural context and regional issues ( Carayannis & Campbell, 2011; European Commission, 2020; Garcia Aracil, 2013; Garcia *et al.*, 2022). In the context of fifth-generation universities, these institutions are responsible not only for the qualification of students, research and knowledge creation, university-industry collaboration for knowledge commercialization for responding to the demands and needs of society but also for the engagement of their activities in economic, social and natural contexts ( Audretsch & Belitski, 2021; European Commission, 2020; Thomas *et al.*, 2023). In these circumstances, universities are increasingly concerned about the importance of strategic planning for value creation. Dahlan *et al.* (2020:47) note that “the role of higher education in society and the economy is changing, and will have to transform the way they deliver their value propositions”. As Carayannis *et al.* (2012:282) state “the emergence of […] quintuple helix structures within society identifies new knowledge production actors within a knowledge-based economy which may signal changes to the university business model value proposition”.

On the other hand, many organisations have traditionally faced a changing context. Still, in recent years international conflicts, financial crises, climate change, technological shifts, political changes, and pandemics have resulted in a highly turbulent environment and an unpredictable future ( Johnsen, 2023). For HEIs, planning strategies in this context is a challenge ( Garcia Aracil, 2013; Thomas *et al.*, 2023; Vlachopoulos, 2021). Thus, Talukdar (2021) notes that HEIs must seek greater agility to cope with technological changes and an increasing amount of environmental disruptiveness. Dahlan *et al.* (2020) highlight some trends that will undoubtedly change how universities operate: the democratization of knowledge and access, contestability of markets and funding, digital technologies, global mobility, and integration with industry. Furthermore, other authors point to some external and internal challenges, which include demographic student changes, increasing higher education demands, increasing competition, international and new emerging models of higher education, society contingencies, increased cost education, lower government funding, rapid technological advances, digitalization, and new entrepreneurial/ethics/sustainable/social standards ( Garcia *et al.*, 2022; Guilbault, 2016). This complexity pulls and pushes universities to experiment with new business models to respond to external and internal challenges ( Audretsch & Belitski, 2021; Dahlan *et al.*, 2020). As stated by Dahlan *et al.* (2020:49), “There is a need for innovative and dynamic business model designs in meeting the changing requirements of the University of the Future”. The study of how universities propose different business models to create different value propositions is an opportunity, since, as Miller *et al.* (2014:266) point out, “the core value offering of a university presents an underresearched context in relation to business model design”.

Most HEIs have shown isomorphism in developing strategic planning according to the same scheme ( Bryson & Alston, 2011). Furthermore, we found that in the process of strategic management of universities traditional strategic tools present some limitations ( Fumasoli & Hladchenko, 2023; Han & Zhong, 2015; Zechlin, 2010). Firstly, they are static ( Fuertes *et al.*, 2020). strategic plans tend to work with long-term goals and are updated after this period, which implies inertia and little flexibility and dynamism. As Johnsen (2023:446) points out, in long-range plans “conditions will often change during the implementation of plans”. Secondly, these strategic plans have been designed by top management, resulting in plans with little participation, which means that implementation is sometimes not very well supported. Thirdly, one-size-fits-all tools are inappropriate. while many challenges are shared among universities, each institution must specify its way of addressing them, *i.e.,* what kind of university it wants to be, what its value is, and what its strategic model will be, questions that are difficult to answer with an isomorphic strategic formulation. On the other hand, the strategic plan entails performance or output goals ( Jalal & Murray, 2019; Zechlin, 2010), without considering other value propositions and other outcomes, monetary or otherwise, that universities can achieve. Finally, Johnsen (2023:446) states that “when there is great uncertainty, authorities and senior managers do not have enough knowledge to make good, long range plans”. According to these authors, some planning tools are too simple to capture the many aspects of reality. In sum, traditional strategic tools are not able to adequately address the whole new circumstances of HEIs, marked by the complexity of their functions and social and economic challenges. Consequently, new strategic tools are needed to define dynamic models ( Garcia Aracil, 2013; Vlachopoulos, 2021). As Audretsch & Belitski (2021:978) suggest, university strategic management requires the application of strategic tools already tested and legitimized in the business context. 

To gain competitive advantages, new more interactive, collaborative, open, and complex business models, with more activities, stakeholders, and mechanisms are required ( Miller *et al*., 2014). Design thinking approach and business modeling tools such as business model environmental map, business model innovation, business model canvas, and value proposition canvas are emerging in the context of universities ( Dahlan *et al.*, 2020). Specifically, the business model canvas (BMC) of Osterwalder and Pigneur (2010) is proposed. It is built on design thinking and joint inquiry techniques and enables the design of new business models in the HEIs. This visual tool allows management practitioners to jointly inquire into specific strategic management problems ( Avdiji *et al.*, 2020). Osterwalder and Pigneur (2010) characterize a BMC by nine key strategic components, In a single-page view. BMC provides an easy, visual, dynamic, intuitive, didactic, and flexible device that can be developed and continuously improved to shape and renew any business strategy. BMC can also highlight the alignment of top strategies and bottom actions, which enhance strategic competitiveness and even discover previously unseen opportunities for value creation. For all these reasons, it is a conceptual tool that many scholars and practitioners have widely adopted for supporting business model innovation, being used in different sectors, for other types of companies, and to explain different performances. There are adaptations of this tool, such as the lean canvas, the ecocanvas, the social canvas, and the sustainable business canvas.

In the context of HEIs, BMC has been timidly used, but either in a partial way or to achieve some specific strategic objectives ( Al-Filali *et al.*, 2024; Dahlan *et al.*, 2020; Gaus & Raith, 2013; Gaus & Raith, 2016; Gomes *et al.*, 2020; Hadăr *et al.*, 2016; Jongbloed & Kottmann, 2022; MacBryde & Franco-Santos, 2016; Mulyana *et al.*, 2018; Polat, 2022; Rytkonën & Nenonen, 2014; Trevisan *et al.*, 2022; Trunina *et al.*, 2021). However, its versatility in developing multiple business models in universities has not been highlighted, despite its focus on creating different types of value for an increasing number of beneficiaries.

This research aims to propose a modified version of BMC, named University-Model Canvas (Unicanvas). This proposal is not just an illustration but a discussion of the implications of the changes, trends, and challenges faced by HEIs in the design of their strategic business models. We also reflect theoretically on the peculiarities of each of the blocks in the context of universities to argue the versatility of the tool to design university business models customized to different value propositions and for different beneficiaries. We also reflect on the suitability of Unicanvas as a strategic model innovation tool to reimagine and redesign changes to some or all of the elements by which HEIs create, deliver, and capture value.

The main contribution of the document is to provide a dynamic and visual tool particularly suitable for defining HEI business models. The main advantage of this tool against traditional tools is that allows the design of business models tailored for different universities, diverse activities, and stakeholders. Thus, Unicanvas is suggested for its effectiveness, practicality, and easy-to-use tool design.

The paper is structured as follows. After this introduction, the importance of strategic management for HEIs is discussed. Subsequently, an analysis of the origins and elements of Unicanvas is discussed. Finally, the conclusion, discussion, contributions, implications, and future lines of research are presented.

## Strategic management in Higher Education Institutions

Traditional strategic models and tools such as Porter's Five Forces, BCG growth-share matrix, SWAT, stakeholder analysis, scenario planning, and Strategic group maps carry out the strategic planning and management process analytically and rationally based on deductive and inductive processes leading to organization and planning. However, "moving beyond traditional strategic management approaches, joint inquiry through design thinking techniques has emerged as a valuable approach in strategy making" ( Avdiji *et al.*, 2020: 696). Unlike traditional tools, visual inquiry tools develop a joint process where alternative hypotheses are explored. These tools have a creative and iterative design based on abduction and invention and in design thinking techniques. They work well with ambiguity and uncertainty, are mainly visual, and are based on sketching and prototyping, intensive observation and wondering, and challenging stereotypical perception ( Avdiji *et al.*, 2020). In the university context, design thinking approaches and business modeling tools such as business model environmental maps, business model canvas, and value proposition design canvas are emerging ( Dahlan *et al.*, 2020). Of these, we will focus on BMC, One of the first and best-known visual inquiry tools that have transformed business modeling by providing a design space framed by nine building blocks.

BMC of Osterwalder and Pigneur (2010) is a holistic, visual, simple, dynamic, participative, unbureaucratic, intuitively understandable, and practical tool for the design of the logic of a firm. Nowadays, the BMC is the most commonly applied tool for business model design ( MacBryde & Franco-Santos, 2016). It takes up the nine strategic building blocks for the value creation process transformation. It assigns them to the four most important areas of the company: offer, customers, infrastructure, and financial structure. The nine blocks are Customer Segments, Value Propositions, Customer Relationships, Distribution Channels, Revenue Streams, Key Activities, Key Resources, Key Partners, and Cost Structure.

Although there are precedents for using BMC in the context of HEIs, mainly it constitutes a tool that is taught for entrepreneurship courses in leading universities ( Türko, 2016), it has been timidly used for the strategic management of universities, but either in a partial way or to achieve some specific strategic objectives. For example, Gaus and Raith (2013) developed a general BMC for entrepreneurial universities. Rytkonën and Nenonen (2014) strove to ascertain how BMC could contribute to university campus management. MacBryde and Franco-Santos (2016) defend the use of BMC for university strategic management proposing that departments and centers should be given free rein to design their own "sustainability model" by developing a more appropriate performance measurement system. Hadăr *et al.* (2016) used it for value propositions and growth strategy in the case of technology transfer centers for valorizing intellectual property rights. Gaus and Raith (2016) explain the design of BMC for public and private universities, considering, respectively, students as resources or as customers. Mulyana *et al.* (2018) and Dahlan *et al.* (2020) have combined in the university context BMC with other strategic tools such as SWAT, Environmental Map, and Value Proposition Design Canvas. Others, such as Gomes *et al.* (2020), applied BMC in the context of the digital transformation of HEIs. Trunina *et al.* (2021) developed BMC in a university context for study programs. Jongbloed and Kottmann (2022) have applied to BMC for universities for value proposition analysis. Trevisan *et al.* (2022) used BMC for HEI eco-innovative strategies. Finally, Polat (2022) applied BMC in technoparks analysis. Al-Filali *et al.* (2024) applied it to develop a sustainable financial plan. All these studies highlight the usefulness of the tool due to its applicability to the achievement of different specific objectives, be it entrepreneurship, sustainability, or digitalization, always highlighting the importance of the value proposition defined by these institutions. However, the versatility of this tool for developing different and innovative business models in universities has not been highlighted.

The need to respond to different challenges, and to design different and tailored business models and therefore different value propositions highlights the opportunity of using BMC as a strategic tool that follows the principle of creating, delivering, and capturing value. The application of the BMC in the context of HEIs as a university strategic tool could enable a holistic, agile, and visual picture of their strategic processes to be generated. Also, BMC allows changes or adjustments, which occur very often in a university context, to be implemented easily and quickly. Finally, BMC is a tool characterized by a high degree of participation. With these antecedents, this article proposes Unicanvas as a practical and dynamic tool to address their strategic concerns from a unique holistic point of view.

## Building Unicanvas

### Origins

The definition of a business model is adopted herein, as stated by Osterwalder and Pigneur (2010), according to which it describes the rationale of how an organization creates, delivers, and captures value. BMC involves translating strategic management concerns, such as positioning and goals, into a visual model that deploys how businesses function. This framework enables the business structure and systems to be designed by conceptualizing an organization through three key aspects: how key components and functions are integrated to deliver value, how they are interrelated within the organization and across its supply chain and stakeholder networks, and how the organization generates value, or creates profit, through those interconnections. This visual tool classifies businesses' processes and internal activities into nine strategic and interlinked categories, each representing a building block.

1. The first block symbolizes the heart of any business model, which is that of customer segments.
2. The value proposition includes combining products and services that create value for customer segments.
3. Customer relationships explain the relationship the business wishes to develop with every customer group.
4. Channels describe the paths a company employs to reach its target customers in delivering its desired value proposition.
5. Revenue streams represent the income a company is reaching.
6. The sixth strategic block, key resources, reveals the most critical assets needed.
7. Key activities are the main tasks and actions that should be deployed.
8. The eighth block identifies the key partners in terms of stakeholders and associates.
9. Cost structure gathers the most critical operational expenses and costs.

Although presented sequentially, the definition of block contents for each business is an iterative process. Following the canvas philosophy, the process of fulfilling in the blocks is an iterative process, usually made with post-its where each one represents one idea defined with three or four words. The reflection and decisions taken in one block condition the next one, but at the same time, the reflection on the following blocks can lead to a reconsideration of the initial decision. Blocks are interrelated, meaning that defining the contents of one block affects the contents of others. This, in turn, may require redefining the contents of the preceding ones. The interrelationships of the blocks give it a dynamic character and make it a tool that allows for rapid changes.

### Unicanvas

**Figure 1.**
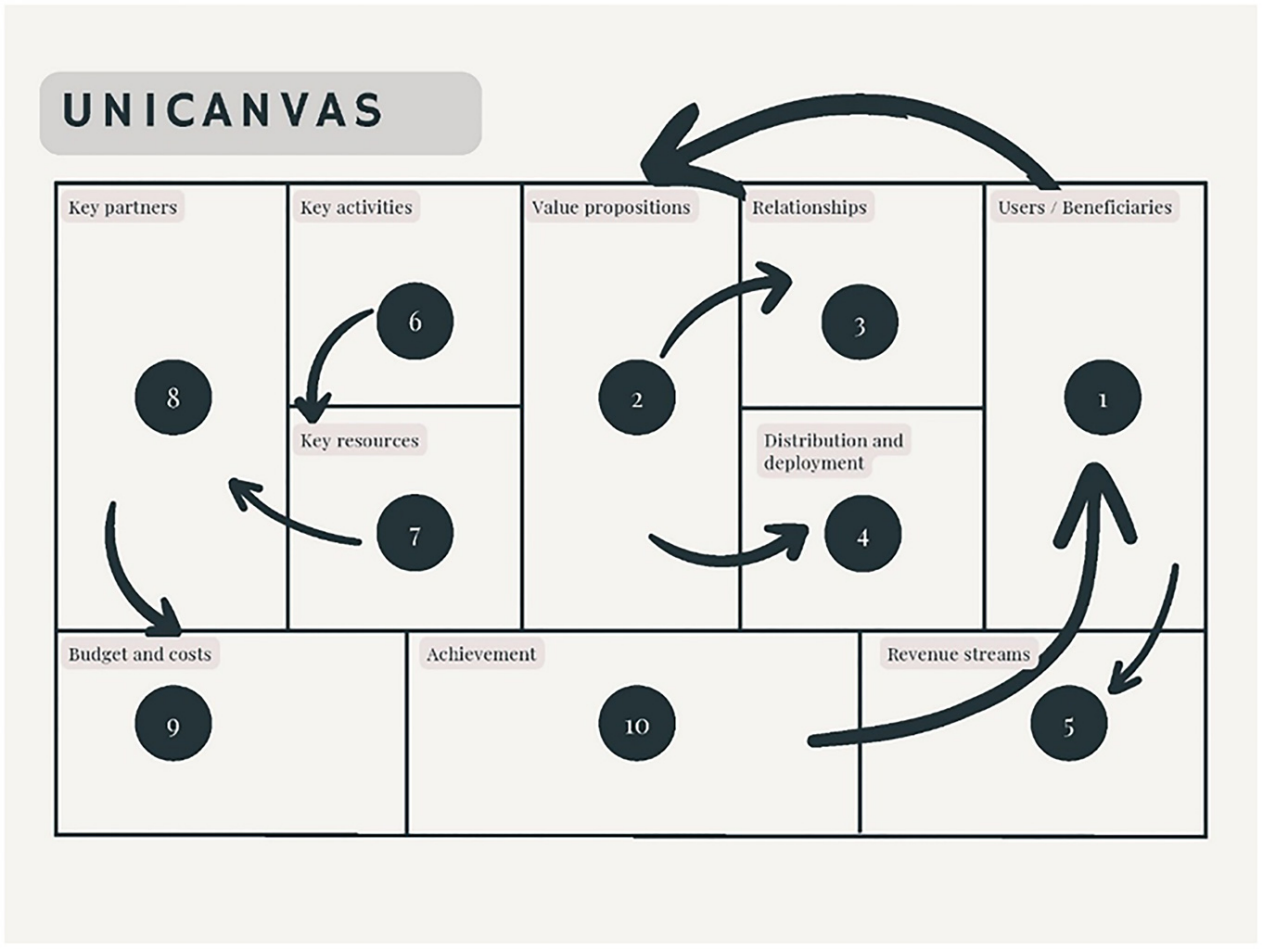
Unicanvas.

The different elements included in the Unicanvas tool are shown in Figure 1.

In the current context of HEIs, Unicanvas allows to establishment of potential scenarios when there are uncertainties associated with the process and to reflect on changes to be undertaken in a stratified manner. Universities could develop alternative Unicanvases to analyze different ideas, routes, or models to reach the agreed vision. Even more, several canvases allow us to analyze different possible business models, looking at their implications, or for different customer segments. Because the proposed blocks are easy to understand, with Unicanvas, the planning process is participatory, and the potential benefits can be integrated into the process. Taking into account these considerations, the process should be developed in the following stages.

First, the key beneficiary identification process precedes the other blocks and is the key to the whole process. This building block answers the question: For whom is value created? ( Osterwalder & Pigneur, 2010). BMC calls this block customer segments, where firms sometimes separate between different segments, and between clients and users.

As in other sectors, and public and private institutions, the marketization of HEIs implies that universities need to become more market-oriented and include a customer orientation in their strategic planning ( Bunce *et al.*, 2017; Guilbault, 2016). This block is key, because as Gaus and Raith (2016:199) state, “the driving force behind sustainable value creation is given by the customer segment(s) responsible for financing the university”. The problem is to identify who are considered the beneficiaries of HEIs. First, because there is an open debate on the role of students. Second, because of the role of universities in the quintuple helix model and the new challenges they face, the number of actors to which universities can add value has expanded, both economically and socially ( Mark, 2013). The first issue, the role of students has been approached differently in public and private universities ( Gaus & Raith, 2016). Gaus and Raith (2013) indicate that it is mainly in private universities where students are viewed as key customers and are offered expert knowledge and a job-qualifying degree as a means of value. In the case of public universities, students can be seen "as (human) resources which are transformed by the education (production) process into qualified professional and, thus, also marketable personnel" ( Gaus & Raith, 2016: 185). Despite this focus on the student as a resource, many assume that even in public universities students are clients because students have a customer's perception and claim the rights observed in the everyday marketplace. Thus, the British government has defined students as customers ( Bunce *et al.*, 2017). And there are authors, as Mark (2013), who argue that universities must continue adopting the student-customer paradigm to increase the quality of their education, the accessibility to the faculty, and the improvement of the efficiency of their processes, opening an interesting debate.

The second issue, HEIs, like other enterprises, have multiple customers and beneficiaries ( Guilbault, 2016), which have been incorporated mainly because of the new roles assumed by fifth-generation universities and the need to generate economic and social value in a challenging environment. Nowadays, the value created by universities is much broader, and the impact of universities on innovation and regional development means that the number of users and beneficiaries is ever-increasing. Gaus and Raith ( 2013; 2016) point out, as beneficiaries, different groups, including firms, start-ups, research institutions, the government (as an employer), the research community, innovative firms, R&D units, and innovative customers. According to Audretsch and Belitski (2021), the roles of all university stakeholders, including university researchers, students, managers, industry, government, venture capitalists, and science park agents could be identified separately. 

The needs and concerns of these potential customers/beneficiaries may diverge significantly, becoming complex and problematic the strategic planning. Unicanvas allows keep in mind and to differentiate each of the beneficiaries based on identifying post-it colors or even making different canvases for each beneficiary. As an illustration of these ideas, and by considering diffuse differences between customer and beneficiary in this context, each university, public or private, should use this block to define its beneficiaries broadly. In this regard, we propose the following potential beneficiaries considering the different sources of value that the fifth-generation university can generate: Students, alumni, and lecturers, in the case of teaching; researchers, academicians, and research centers, for research; academic entrepreneurs, spin-offs and start-ups, external firms, employers, R&D centers, and the government and its administrators, for transfer; and families, agencies, the general public, social groups, professional sectors and society to complete the quintuple helix model. From this wide variety of beneficiaries common to all universities, we propose that, in this block, each university should select only the key beneficiaries to develop the desired future of the institution. Those that will be the focus of its strategy, i.e., different universities could give greater emphasis to one of their missions, and therefore focus more on one type of beneficiary or even different universities may identify the same key beneficiaries, but there may be differences in the specific segment of that beneficiary on which they focus. For example, certain universities located in older populations may focus their strategy on attracting foreign students and these may be their key or try to find more students in the lifelong learning niche. The choice made will condition the decisions and choices of the other blocks.

Second, once the beneficiaries have been identified the next process is the value propositions, precisely targeted at those key beneficiaries. This block answers the questions: What value is offered? What problems are solved? Which needs are fulfilled? Which goods or services are provided? ( Osterwalder & Pigneur, 2010). The core of BMC involves the proposition of the firm's value, which defines the kind of value supplied. In the recent context, HEIs provide complex offers since they arise from teaching, research, and technology transfer and in response to the society and natural environment. HEIs value generation is a mix of teaching/research/transfer or impact on the territory.

Hence, to make a difference, we propose that each university designs its value propositions as a unique combination of products and services that provide differential value to its key various beneficiaries. The traditional aim of universities is teaching, that is, to disseminate knowledge to students to shape them into first-class graduates who will be hired by public or private firms ( Gaus & Raith, 2013). Depending on whether HEIs are funded by tuition fees or by public funding, different factors will be taken into account, such as qualifications leading to employment, valuable education and training opportunities, proximity, location, and physical structures. For other beneficiaries, such as alumni and lecturers, new value propositions can be designed, aimed at lifelong learning and employability. Furthermore, the new offer of micro-credentials, such as long-term continuing education, is an opportunity for a differentiated value proposition. Likewise, for researchers, academicians, and research centers, the proposition of the value of an HEI as a research institution is evaluated by its research infrastructure incentives but also by quality indicators ( *e.g.*, number of citations, research projects, and contracts, and awards), range from publications to patents that document the outcomes of their innovative research. Related to the transfer mission, value is generated from the technology and innovation brought to the market as a result of the commercial exploitation of the research results generated within the University. Among others, spin-offs and start-up creation, R&D transfer, marketable products, services, patents, licenses, contracts and clustering, incubation, acceleration programs, networking opportunities, and Technology Transfer Office (TTO) services. Finally, universities currently must assume a crucial and central role in regional development, key for the government and society, by engaging in new activities to offer economic, social, and cultural contributions and legitimization and recognition. For example, by providing training and knowledge that solve problems in the lives of cities or specific groups ( Mato de la Iglesia & Gutierrez-Solana, 2021). Innovation is also an important value for universities that can materialize in the development of products and services that prioritize the interests of society ( Carayannis & Rakhmatullin, 2014), innovations that are concerned with sustainability ( Machado *et al.,* 2018), and innovations aimed at solving the challenges of global warming and sustainable development.

Third, beneficiary relationships. This block answers the question: What is the relationship with each customer segment? ( Osterwalder & Pigneur, 2010). identifying key beneficiary segments is complex for HEIs. Therefore, the complexity of its strategic management is accentuated when different segments require different relationships because one of the universities' main goals is to develop longstanding value from reciprocally valuable relationships with each beneficiary segment. According to Gaus and Raith (2013), HEIs that consider students as customers could strengthen their relationships through mentoring programs, student organizations, internships, and student programs. Likewise, for students, alumni, and lecturers HEIs develop personal assistance and orientation through assisting, guiding, and mentoring programs. For researchers, academicians, and research centers, HEIs use advising and informing programs and participate in and value co-creation. Internships, TTOs, business incubators, academic conferences, R+D networks, and external theses could be promoted for transfer activities ( Gaus & Raith, 2016). Finally, creating and maintaining communities or ecosystems is key to completing the new mission of universities.

Therefore, each university can design its routines/protocols and describe how it establishes, maintains, and enhances contact with each beneficiary segment, i.e., it should establish activities to attract and maintain beneficiaries, *e.g.*, students, prestigious researchers, and communities. Consequently, strategic communication units are key in establishing and maintaining a good relationship with key beneficiaries and promoting the programs and services provided, *e.g.*, websites, social media, mailing, or fairs and exhibitions.

Fourth, how distribution and deployment channels are established. This block answers the question: Which are the main distribution channels? ( Osterwalder & Pigneur, 2010). Value propositions of universities are delivered through different distribution channels to their beneficiaries ( MacBryde & Franco-Santos, 2016). According to Gaus and Raith ( 2013; 2016), universities must develop job platforms, and courses, seminars, journals, publishers, calls for proposals, start-ups, academic spin-offs, and transfer units. Gaus and Raith (2016:184) indicated that “value distribution by the market-oriented university addresses a new customer segment with which the university can generate monetary revenues that change its income structure quantitatively as well as qualitatively”

In 2016, Steve Blank created Mission Model Canvas, an alternative to the BMC for military units. This variant proposes relabelling this block as deployment instead of distribution. Following his arguments, in the business world, we should ask: What type of distribution channel do we choose to deliver the product/service from our company to the customer segments? However, for the US Department of Defense, Blank (2016) proposes: How can we deploy the product/service for widespread use among people who need it? What channels can we use, and how can we communicate our value proposition to our beneficiaries? In this direction, it is necessary to define some key aspects, such as establishing the message distribution channels, outlining a promotion plan, or identifying the customer segment involved in the action.

For universities, this block involves getting in touch with their beneficiaries, delivering their value proposition, and creating the beneficiary experience ( Osterwalder & Pigneur, 2010). The choice of appropriate channels for each beneficiary segment and the effective use of channels deliver and raise awareness of the services provided. each university should design its distribution and deployment channels and describe the service distribution and communication channels needed to present its value to its beneficiaries. Depending on the chosen key beneficiaries, some channels will be more strategic than others. Examples of channels are educational programs and events organized by HEIs or in which they participate, such as training courses, face-to-face and online classes, seminars, conferences, meetings, competitions, business conferences, networking and cultural events, intranet tools, e-learning, instant messaging, voice and video conferences, meetings, seminars, conferences and congresses, hackathons, document collaboration tools, task management tools, networking events, popular books, audiovisual exhibitions, apps, brochures, blogs, shows or media. Not all universities should give the same importance to all channels.

The fifth block, revenue streams, answers the question: Which values are being paid for? How are payments made? What are the relative shares of individual revenue streams? ( Osterwalder & Pigneur, 2010). This block describes the available sources of funding, the budget, and the financial set-up needed to deliver the value proposition.

 Traditionally, public universities have been dependent on public funding from governments and their main objective has not been to generate income. As Gaus and Raith (2016) state, a public university model, that is fully funded by the government, is not the same as a private university without government funding. However, in the current context, with reliance on tuition fees, inflation, higher prices, inadequate facilities, increasingly acute funding problems, political conflicts, The growing cost of the system, and facing some trends such as strong competition or demographic change, not only private but also public universities need to generate new sources of income and diversify revenues streams ( Al-Filali *et al*., 2024). This has forced developed countries to change their public university incomes, which were initially dependent only on public financial support. In conclusion, Public universities are also encouraged to find new sources of revenue through activities that place them in the knowledge-based economy.

In this respect, Mulyana *et al.* (2018) identified a business model development strategy for Padjadjaran University, to alter their general university management to be more creative in finding other revenue streams to maintain their sustainability. As Gaus and Raith (2013) argue, each university should indicate various sources of income, with different percentage weights, not only in the form of student fees, research grants, and public government funds but also as transfer income, such as equity stakes, patent sales, licensing and contract income, service income and product sales. In their literature review, Al-Filali *et al.* (2024) identify traditional sources of income as tuition fees, fees, and government funding, and others as research consultancy and industrial training, building rentals, laboratory services, scientific consultancy, research grants, endowments, housing fees, contracts, ancillary services, and philanthropy. Consequently, we propose that each university should indicate its strategic pathways to financial flows and the sustainability of its model. Each HEI could establish its revenue streams from rental fees (conference rooms, land, university residences, and residence hall), property management, and fees of services. 

In sixth place, key activities should be defined. This block answers the question: Which activities for value creation are required? ( Osterwalder & Pigneur, 2010). According to Gaus and Raith (2013), universities could develop key activities, such as teaching, research, transfer, proactive market participation, and product development. Recently, university challenges have contributed to the deployment of new activities, such as social responsibility, sustainability, entrepreneurship, and cultural and regional development.

Considering the contents of prior building blocks, each university should describe how its key activities are organized by providing the initiatives required to generate value for its beneficiaries/users. Consequently, Unicanvas could consider key activities such as administration, property management, facilities management, corporate communication, marketing and networking, entrepreneur support, technologies assisting, incubation programs, industry-university cooperation, contracts, IPR licensing, and cultural and dissemination activities. the selection carried out in the previous blocks determines the key activities. For example, a university that has chosen to internationalize its students through agreements with international universities should prioritize the establishment of agreements or one that chooses to become a lifelong university may prioritize e-learning.

Seven, key resources answer the question: Which resources are required for value creation? ( Osterwalder & Pigneur, 2010). Various key resources for value creation, such as teaching staff, students as input, senior and junior scientists, a complete research infrastructure, research findings, researchers, and transfer experts, can be found. These resources are supported by the university infrastructure and administration, technological support, science parks, academic and business networks, university incubators, and efficient TTOs ( Gaus & Raith, 2013).

We also suggest that universities describe their critical resources, whether physical or intangible, to offer value to their beneficiaries. Human resources, talent, knowledge, technological resources (laboratories or equipment), financial resources, and credibility are all essential for universities. But also, key properties, financial resources, management competencies, and even image and reputation. Depending on its value proposition, some resources will be more relevant than others. For example, for a university that wants to reach a dispersed territory, its key resources will be different if the teaching is face-to-face or online. The resource and activities blocks need to ensure the viability of the model in the digital era, developing digital platforms, talent, cyber-security, and lean management.

Eight, key partnerships answer the question: Who are the most important partners for value creation? ( Osterwalder & Pigneur, 2010). Value creation also requires the support of a national and international network of associates to produce and deliver this value. According to Gaus and Raith (2013), Stolze (2021), and Audretsch and Belitski (2021), partners could include not only teaching staff, firms, public institutions, and researchers from other universities or research centers but also business incubators, scientific parks, venture capitalists, business angels, technology transfer services, entrepreneurship centers services, private and not-for-profit firms and organizations, and, of course, alumni. In this regard, Unicanvas proposes that universities require the establishment of long-term and closer relationships with partners from different contexts.

HEIs should describe their network of cooperation agreements that seek to deliver higher values and could include other potential partnerships. Unicanvas could help by identifying current partnerships and disclosing potential external clients for future contacts. Other universities, research institutions, government institutions, public and private agencies, local government, cluster, and organized industrial areas, chambers of commerce, business associations, and technoparks should be considered. For example, if we have chosen dual programs as a key strategy, one of our key partnerships should be companies and institutions that offer work experiences and internships.

Nine, budget and cost structure. This block answers the questions: Which are the most important expenditures? Which activities/resources create the highest costs? ( Osterwalder & Pigneur, 2010). The strategic plan includes the final costs derived from its structure and means involved in the operation of the model. In this respect, Gaus and Raith (2013) describe the personnel expenses (such as salaries of academic and administrative personnel) and the infrastructure and administration costs. Cost is a common major concern affecting all universities. HEIs deploy programs to reduce bureaucratization, optimize resources, promote ROI-driven culture, back office outsourcing, and cost efficiency ( Dahlan *et al.*, 2020). Furthermore, in addition to financial costs, ecological and social costs of universities should be included.

Similarly, the mission model canvas proposes that organizational budgets should match the cost structure. In this respect, each solution needs to be evaluated based on the budget of the required solution ( Blank, 2016). To depict these arguments, universities need to identify the main costs associated with this model, including financial, environmental, and social impacts, as well as their budget. Consequently, each university could consider initial, maintenance, and operational costs.

Finally, achievement block. To the strategic building blocks of BMC, we propose to add a block in the Unicanvas. In his mission model canvas, Blank (2016) argued that the revenue streams block makes no sense in this setting and relabelled the previous block as mission achievement/success. Blank defines achievement as the global value created for the sum of all of the beneficiaries/the greater good, which can be measured in a variety of ways, but all deliver value to the end beneficiary. We propose that this new block can be more suitable for collecting data for strategic planning at universities.

In the university context, mainly in public HEIS, the term 'business' or 'revenue streams' can be questionable as the overall mission because the majority of universities do not involve making a profit, as argued by MacBryde and Franco-Santos (2016). The way to capture value can come through other ways such as reputational benefit. Gaus and Raith (2013) note that students also generate a reputational benefit (non-monetary). These authors highlight that in addition to revenues, students and their insertion into the labor market generate a reputational benefit for universities; similarly, reputation can be increased through the impact of research and its influence on research rankings, journal publications/editorships, and the number of patents o the image that the university projects in its surroundings through contracts, R&D transfer, spin-offs or cultural events. All these achievements will undoubtedly have an impact on the revenue stream block for those universities that are well-positioned in these rankings. Thus, Vidal and Ferreira (2020) point out that the reputation gained from the rankings can influence the income obtained by the universities, through enrolments or contracts. For small universities that do not normally rank highly, this effect is not relevant.

Furthermore, universities can also have an impact on society by making it more educated, as Mato de la Iglesia and Gutierrez-Solana (2021) argue. According to these authors, some of these effects are related to the tractive function in the territory or within the university community, to the consolidation of the university's public image, as well as other types of impacts related, for example, to the improvement in the processes of accessibility to academic and university knowledge for disadvantaged groups or to cooperation for development and the improvement of opportunities in socially disadvantaged communities. Within this block, measures of social achievements such as inclusion, equity, equality, development cooperation, social responsibility, culture, scientific dissemination, and professional dissemination could be included. For example, its impact on variables related to the progress of culture, science, and the democratic state, its impact on development aligned with the interests of society, or its impact on the innovative and sustainable development of the territory, or the productive sector.

This added block therefore explicitly includes non-monetary benefits or revenue streams produced in all universities. in the Unicanvas model, we propose that each university could have its own 'achievement block' depending on its business model and approaches derived from its vision. In response to the new challenges facing all types of public and private universities today, any university must pursue both types of benefits (monetary and non-monetary) to a greater or lesser extent, and the cohesion of the latter two blocks in Univanvas makes it possible to expressly take them into account in the reflections and innovations of university business models 

## Conclusions

HEIs are facing significant challenges that require their business models to be designed with more agile, visual practices, intuitive and dynamic, and less bureaucratic and static tools. Traditional strategic models and tools, criticized for their static and isomorphic nature, need to evolve towards models focused on creating, delivering, and capturing value, such as BMC. Universities need innovative strategic business models that continuously fit with relevant stakeholders, and need innovative and dynamic tools in meeting their changing requirements.

The paper's main contribution is that Unicanvas provides a starting point for the consideration, discussion, and transformation of the logic of strategic management in HEIs and how it should be delivered and captured. Unicanvas could facilitate the distribution of strategic management knowledge (and its key drivers), enhance the understanding of competitive variables, and improve activity choices, resource allocation, and decision-making.

Encouraging the participation of the different actors involved in the process, and accompanied by other strategic tools, Unicanvas could facilitate the integration of the debate of the different stakeholders involved in the reflection. Strategic planning is normally considered the main element, but not the essence, of strategic management, which instead uses several phases considered outside of the realm of planning (in the strictest sense of the term) that are related instead to resource management, the implementation of activities and processes, and control and evaluation. Unicanvas helps the definition of key performance indicators, as it facilitates their evolution to the Balanced Score Card ( Sánchez Vázquez *et al.*, 2016). Furthermore, Unicanvas simplifies the implementation of the strategy, defining key activities, resources, allies, and costs, addressing one of the main problems for strategic management, especially in universities, where strategic plans are often established for four or five years that are neither developed nor followed up.

Some theoretical implications can be derived from our proposal. First, Unicanvas, specifically the identification of the beneficiaries’ segment, could enrich the focus of the Stakeholders Theory by better identifying beneficiaries and key partnerships blocks through the process of mutual value exchange beyond the process of resource transaction ( Falqueto *et al.*, 2020; Freudenreich *et al.*, 2020; Miller *et al*., 2014; Polat, 2022; Stolze, 2021), considering the ecosystem and actively managing the HEIs'transformation process ( Stolze, 2021). Second, blocks of key partners, resources, and activities could be underpinned by the Resource-based View. This theoretical approach would help to classify the main value-generating resources of HEIs. Third, Network Theory could shed light on identifying the relationship and key partnerships blocks. Finally, Institutional Theory ( Fernández-Alles & Valle-Cabrera, 2006; Meyer & Rowan, 2012; North, 1990) with its formal and informal factors and their different pressures, should be taken into account in the design of Unicanvas. This theory emphasizes the role of institutions in economic development and has proved to be one of the most appropriate frameworks for the analysis of institutional factors ( Guerrero-Cano *et al.* 2006; Guerrero-Cano, 2008). Institutional Theory could show that universities from the same institutional context will share some blocks. Likewise, different institutional contexts, and different endogenous and exogenous forces, would explain different strategic business models in universities. As Gupta *et al*. (2023) suggest, each country has different educational regimes and hierarchies, university fees, grants, and scholarships. As Stolze (2021) states, "higher education is highly regulated" and is strongly influenced by the changing context. This theory also could help to consider the entrepreneurial ecosystem that is currently so important for the new challenges of universities ( Audretsch & Belitski, 2021).

This research has practical implications, encouraging administrators to develop visual thinking for business model innovation of HEIs. Unicanvas differs ( Jalal & Murray, 2019) from the traditional strategic planning models and tools applied in most universities, which have been criticized and limited by their static nature, by their implementation problems due to the low participation of key agents in their development, and by their limited capacity to adapt to universities in different contexts and with different orientations. Unicanvas seeks to improve understanding of the particular characteristics of each university, enabling a holistic assessment of changes or proposals in successful models and is ideal for easily gathering key aspects of the model and communicating them, enabling a rapid answer to contingency factors and global disruptions. Unicanvas helps to formulate, design, and validate proposals before they are implemented, allowing prototyping and experimentation. Those responsible for university management should think in terms of value creation and reflect in a particular way on each of the blocks of the Unicanvas, assuming that their institutions' roles are increasingly diverse, and the complexity of management is increasing.

This does not mean that it is the only tool to be used in a complex institution, such as HEIs. Despite its advantages, the BMC, and therefore the proposed tool Unicanvas, is not without its limitations ( Coes, 2014). For example, although the unicanvas includes and enhances a reflection on customers/beneficiaries (stakeholders), or key resources, it does not provide a complete framework for analyzing the external and internal environment in detail. Complementary analyses are crucial to understanding how external trends and factors (legal, demographic, technological changes, etc.) or internal factors (inimitable resources, capabilities, etc.) can impact value generation, generating opportunities or sources of strategic uncertainties. To deepen the strategic analysis, it should be completed with, for example, a Stakeholder Analysis to help practitioners in examining the impact of multiple stakeholders' power, and influence on business model innovation ( Miller *et al.*, 2014). This development provides a new perspective beyond the traditional cost/revenue model architecture. Likewise, PEST analysis or VRIO analysis identifies threats or opportunities, strengths, and weaknesses, but also the sources of strategic uncertainty to assess them and establish Scenario Planning to reduce the risks associated with such uncertainty. Other complementary tools can be casual mapping and we suggest, in line with MacBryde and Franco-Santos (2016), that using Unicanvas can help universities to develop more and better strategic performance measures.

Also, Unicanvas would have some implications in the HEI context for policymakers by allowing them to influence the design of an institutional framework to improve the functioning of universities and their value creation. Future research should also be oriented towards gathering further proposals for initiating the design of Unicanvas starting from the mission or value proposition of HEIs, and towards its empirical application to the specific case of a university or consortium of universities. Furthermore, future lines of research aimed at the practical application of unicanvas for concrete cases, explaining the design process, its dynamics of creation, and how the different blocks are completed.

## Ethics and consent

Ethical approval and consent were not required.

## Data Availability

No data are associated with this article.
