# Supplementary material for: Unicanvas: Exploring a tool for strategic management
Source: Open Res Eur. 2024 Dec 5;4:64. Originally published 2024 Apr 11. [Version 3] doi: 10.12688/openreseurope.17233.3 (PMC11179048; doi:10.12688/openreseurope.17233.3)
Supplement: Peer review history

# Reviewer report: Reviewer response for version 3

**DOI:** 10.21956/openreseurope.20657.r48972

**Published:** 2025-01-23

**Authors:** Edoardo Ongaro

**Affiliations:** 1 The Open University, Milton Keynes, UK

The paper in its version 3 is very clear and makes the argument effectively. It provides a well focused contribution to the important topic/issue of the strategic management of HEIs. The successive revisions (from what I could understand of the process from reading here, as I am reviewing the paper first time) have clearly further sharpened the argument and the flow of the text, and the conclusion section clearly outlines limitations and areas for future research. 

 In formulating a few recommendations / areas for possible further revision, I am concentrating on the following points, which might possibly be discussed or at least acknowledged in the paper, should the authors agree. First, the approach - Unicanvas tool - outlined in the paper might seem to omit the dimensions of power and conflict: universities are arenas where different stakeholders (which are fully recognised and encompassed by the model proposed) may engage in relations which may be competitive or outright conflictual as well as collaborative. For example, managers/administrators, on one hand, and academics, on the other, may have different priorities, views, and indeed visions of what a university stands for - an aspect which may complicate the usage of the Unicanvas tool, and which could usefully be recognised to enable utilising it. 

 Indeed, and this relates to the second point (also acknowledged in a previous reviewer's report), HEIs operate in very different contexts (context being 'larger and deeper' than the organisational environment - e.g. - and with apologies for a self-citation - this is discussed in chapter 7 in Ferlie and Ongaro, 2022). This can have a higher order influence on decision-making (hence also strategy-making): for example, in some countries HEI administrators are mostly academics from within the institution who serve as administrators in elective roles, while in other countries they are 'professional managers' of the Higher education sector appointed by the board or similar organ, and many of them with a non-academic background. This can determine very different decision dynamics. Contextual influences may shape the very premises of strategy-making for a public service organisation (higher education is generally considered a public service, in which both public and private organisations, in terms of legal basis, partake). This is acknowledged by the paper where it says that HEIs in similar contexts may be similar in relation to certain blocks - but this may go deeper that just resemblance and may require being considered more explicitly.

 Third and final (and this too already referred to in previous reviewers' reports), universities are here assumed to be responsive to stakeholders demands, but what about the role of universities in fostering critical thinking and proposing new theories and frameworks to analyse reality? Responsiveness - as noted in the paper already - can entail isomorphism, but this may lead to HEIs missing out on an important part of their (traditional? Historical?) mission: to operate as pathmakers for new lines of thinking and inquiry. This point too might deserve being considered by the paper. 

Is the argument persuasive and supported by appropriate evidence?

Yes

Is the work clearly and cogently presented?

Yes

Is the topic of the essay discussed accurately in the context of the current literature?

Yes

Does the essay contribute to the cultural, historical, social understanding of the field?

Partly

Reviewer Expertise:

Strategic management of public service organisation; public management; public administration

I confirm that I have read this submission and believe that I have an appropriate level of expertise to confirm that it is of an acceptable scientific standard, however I have significant reservations, as outlined above.

## References

[1] : Strategic Management in Public Services Organizations.2022; 10.4324/9781003054917 10.4324/9781003054917

# Response

**Published:** 2025-01-23

**Authors:** Jose M. Sanchez

**Affiliations:** Business organization, Universidad de Cadiz, Cádiz, Andalusia, Spain

Dear Professor Ongaro, Thank you for your efforts to improve our Unicanvas proposal. The three points you mentioned are of great interest. As we have already mentioned to a previous reviewer, we will consider these proposals for improvement in works, that we are carrying out with empirical evidence, and which we hope will be published in international journals. First, we fully agree that we need to incorporate tensions, conflict, and power. This point is emerging in the empirical studies we are carrying out to analyse how the tool can be used. I can tell you that, at least for the moment, this aspect does not complicate the use of the tool per se, but the filling in of the different boxes does generate tensions and discussions (perhaps necessary, in some cases, don't you think?).

 Regarding the second point, it is necessary to adapt the tool to different contexts. This article is a first proposal, and we advocate that all tools should be adapted to each organisation, and not the other way around (as some consultants some>mes try to do ...some>mes).

 Finally, we agree. We can never lose our freedom and stop being a meeting point for the generation of critical thinking. Perhaps we should explain better, in future works, that the strategic definition of an HEI should reflect its positioning and defend its role as a creator of thought.

 We would like to reiterate our thanks for all your sugges>ons. Thank you very much!

# Reviewer report: Reviewer response for version 3

**DOI:** 10.21956/openreseurope.20657.r48162

**Published:** 2025-01-15

**Authors:** Tommaso Minola

**Affiliations:** 1 University of Bergamo, Bergamo, Italy

**Specific Comments**

 Overall, the document has shown substantial improvement in terms of theoretical robustness, coherence, and clarity. Below, I provide a detailed evaluation of how my previous suggestions have been addressed in the updated version.

**First Suggestion:**

 The inclusion of the recommended literature on entrepreneurial universities and business model innovation, as well as the additional references suggested by other reviewers, has significantly strengthened the theoretical arguments supporting the essay’s contribution (i.e. limitations of existing strategic planning tools and the diverse stakeholders universities must address).

**Second Suggestion:**

 I appreciate the clarification that the application of a hypothetical HEI using the Unicanvas framework is intended as further research. As another reviewer suggested, incorporating a case study that explores the challenges of implementing Unicanvas would add considerable value. Such a case study could serve as a valuable reference for HEI administrators to ensure the effective application of the tool.

**Third Suggestion:**

 The expanded explanation of the "achievement block," supported by clear examples of potential measures and variables, has enhanced the section significantly. The discussion also effectively links the "achievement" to the "revenue stream" block, highlighting this feature as a key differentiation of the Unicanvas proposal. Furthermore, the arguments for retaining the label “revenue streams” were convincing, particularly the reasoning that while it may raise questions for public universities, it remains a necessary component.

**Fourth Recommendation:**

 The explanation of how Institutional Theory can be used along the Unicanvas tool is now better substantiated with citations and specific arguments, providing stronger support for this aspect of the essay.

**General Observations:**

 I reviewed all comments from other reviewers and noted that, together with my suggestions, the authors have addressed most points in a thoughtful and effective manner. They have prioritized aligning and focusing the various HEI perspectives (i.e. quintuple helix), resulting in a more streamlined and compact Unicanvas proposal compared to earlier versions. This is remarkable, given the diverse perspectives offered by the reviewers.

**Final Suggestions:**

 While the essay has improved significantly, I have two additional recommendations that could further strengthen the work:

 1. **Include a Brief Description of the Development Process for Unicanvas:**

 One of the reviewers suggested including a short explanation of the process followed to develop Unicanvas. While the authors provided this explanation in their response to the reviewer who asked for it, it is not currently included in the essay. Adding this description to the "Building Unicanvas" section, within the “Origins” subsection (page 3) could provide valuable context and enhance the credibility of the tool.

 2. **Address Minor Typos:**

 Throughout the document, there are some typographical errors, particularly involving inconsistent use of uppercase and lowercase letters after punctuation marks. Examples include:

 o    Page 3, fourth paragraph, the phrase beginning with “strategic plans.”

 o    Page 3, fourth paragraph, the phrase starting with “while means ….”

 I recommend reviewing the entire document to correct such issues.

Is the argument persuasive and supported by appropriate evidence?

Partly

Is the work clearly and cogently presented?

Yes

Is the topic of the essay discussed accurately in the context of the current literature?

Partly

Does the essay contribute to the cultural, historical, social understanding of the field?

Yes

Reviewer Expertise:

N/A

I confirm that I have read this submission and believe that I have an appropriate level of expertise to confirm that it is of an acceptable scientific standard.

# Reviewer report: Reviewer response for version 3

**DOI:** 10.21956/openreseurope.20657.r48217

**Published:** 2024-12-20

**Authors:** Bahtiar Mohamad

**Affiliations:** 1 Universiti Utara Malaysia, Kuala Lumpur, Malaysia

The article introduces **Unicanvas**, a strategic management tool designed specifically for higher education institutions (HEIs). Building on established methodologies like the Business Model Canvas, the framework is tailored to address the unique needs and complexities of HEIs. The authors outline the origins of Unicanvas, its core components, and its potential applications in university management. Furthermore, the article situates Unicanvas within the broader context of strategic planning, entrepreneurial universities, and innovation models, emphasizing its relevance as a holistic tool for fostering institutional innovation, improving performance, and promoting sustainability.

 Drawing from a diverse range of sources, including the Quintuple Helix model and sustainable business strategies, the article provides a robust theoretical foundation for Unicanvas. Key references on entrepreneurial universities and HEI strategic management are seamlessly integrated, supporting the framework's relevance. However, the discussion overlooks critical contemporary research on digital transformation and agile management in HEIs, particularly post-2021 studies. Incorporating these recent developments, including post-pandemic shifts and the digitalization of HEIs, would enhance the framework’s applicability and relevance. Additionally, exploring how Unicanvas complements or contrasts with emerging tools like HEInnovate could further strengthen the discussion.

 The article follows a logical structure, moving from an introduction to the framework’s explanation, application, and conclusion. Visual aids, such as Figure 1, effectively support comprehension. However, the narrative occasionally assumes prior knowledge of strategic management concepts, potentially alienating readers less familiar with the field. Sections like the "Origins" of Unicanvas are overly descriptive and lack critical analysis. To address this, the authors could provide brief explanations of key concepts (e.g., Business Model Canvas, Quintuple Helix) and critically evaluate why existing frameworks fall short for HEIs, thereby highlighting Unicanvas's distinctive contributions.

 Although Unicanvas is presented as a theoretically sound and practical tool, the argument is weakened by a lack of empirical validation. The claims rely heavily on theoretical connections, with limited evidence of its real-world impact. Including detailed case studies or pilot projects demonstrating Unicanvas’s implementation would significantly bolster its credibility. Quantitative or qualitative data illustrating its effectiveness in driving innovation or enhancing institutional performance would further substantiate the authors' claims.

 By aligning Unicanvas with sustainability and societal goals, such as those outlined in the Quintuple Helix model, the article positions HEIs as critical agents of global change. However, the cultural dimension remains underexplored, particularly regarding how Unicanvas can be adapted to diverse institutional, cultural, or regional contexts. Providing examples of such adaptations and discussing the framework’s role in supporting equity, inclusivity, and cultural sustainability would enhance its impact.

Is the argument persuasive and supported by appropriate evidence?

Yes

Is the work clearly and cogently presented?

Yes

Is the topic of the essay discussed accurately in the context of the current literature?

Yes

Does the essay contribute to the cultural, historical, social understanding of the field?

Yes

Reviewer Expertise:

Business and management

I confirm that I have read this submission and believe that I have an appropriate level of expertise to confirm that it is of an acceptable scientific standard, however I have significant reservations, as outlined above.

# Response

**Published:** 2025-01-07

**Authors:** Jose M. Sanchez

**Affiliations:** Business organization, Universidad de Cadiz, Cádiz, Andalusia, Spain

Thank you very much for your review. We have completed a detailed reading of all your comments and deeply appreciate your suggestions. Indeed, digital transformation is an aspect that we will consider in future work. Our university is facing this structural change. Regarding the HEInnovate tools, we are already using them in other lines of research, and it may be very interesting and timely to incorporate them in this one. We thank you for your comment, and in future works we will explain in more detail key concepts (e.g. Business Model Canvas, Quintuple Helix). Indeed, we are in the process of empirical validation. We have already done one case, but we want to expand the number of cases to capture the richness and diversity of universities that at first glance may seem similar, including the cultural dimension, as you suggest. Thanks ¡!

# Reviewer report: Reviewer response for version 2

**DOI:** 10.21956/openreseurope.19793.r44063

**Published:** 2024-10-25

**Authors:** Rafael Ventura

**Affiliations:** 1 University of Malaga, Málaga, Spain

The authors have satisfactorily addressed the suggestions I made in the first revision. The new version has corrected the weaknesses of the first one. The new version is significantly better than the first and addresses the main weaknesses of the first version.

 I do appreciate the practical contributions of this work, congratulating the authors for their contribution.

 I highlight the usefulness of the results for decision-making in university management.

Is the argument persuasive and supported by appropriate evidence?

Partly

Is the work clearly and cogently presented?

Yes

Is the topic of the essay discussed accurately in the context of the current literature?

Yes

Does the essay contribute to the cultural, historical, social understanding of the field?

Partly

Reviewer Expertise:

Business Model Innovation.

I confirm that I have read this submission and believe that I have an appropriate level of expertise to confirm that it is of an acceptable scientific standard.

# Reviewer report: Reviewer response for version 2

**DOI:** 10.21956/openreseurope.19793.r44067

**Published:** 2024-10-18

**Authors:** Ursula Weisenfeld

**Affiliations:** 1 Leuphana University, Lüneburg, Germany

The authors have implemented appropriate changes to the manuscript and have further developed Unicanvas as a useful tool for HEO to navigate complexity.

 I beg to disagree with the conclusion: “universities must continue adopting the student-customer paradigm to increase the quality of their education” (Indeed, one might suggest that ‘students as customers’ decreases the quality of education – thus, I would phrase this more cautiously).

 There are some formal aspects to be considered, such as some incorrect wording (for example “Unicanvas allows to establishment of potential scenarios”; (p.5) and some repetitions, such as

 “a tool that is taught for entrepreneurship courses in the leading universities (Türko, 2016), BMC has been timidly used, but either in a partial way or to achieve some specific strategic objectives” (p. 4 and repeated on p. 5)

Is the argument persuasive and supported by appropriate evidence?

Partly

Is the work clearly and cogently presented?

Yes

Is the topic of the essay discussed accurately in the context of the current literature?

Yes

Does the essay contribute to the cultural, historical, social understanding of the field?

Partly

Reviewer Expertise:

innovation and change

I confirm that I have read this submission and believe that I have an appropriate level of expertise to confirm that it is of an acceptable scientific standard.

# Response

**Published:** 2024-10-19

**Authors:** Jose M. Sanchez

**Affiliations:** Business organization, Universidad de Cadiz, Cádiz, Andalusia, Spain

Thank you very much, professor Weisenfeld, we will incorporate your comments in the next version. Thank you very much for your work and help.

# Reviewer report: Reviewer response for version 2

**DOI:** 10.21956/openreseurope.19793.r44066

**Published:** 2024-10-10

**Authors:** Jasmina Berbegal-Mirabent

**Affiliations:** 1 Universitat Politecnica de Catalunya, Barcelona, Catalonia, Spain

I appreciate the authors' effort in improving their manuscript in response to the comments raised by the reviewers. The revised version shows a significant improvement, demonstrating a more robust and comprehensive approach. I have no further comments or suggestions. Congratulations on this interesting and well-developed piece of work.

Is the argument persuasive and supported by appropriate evidence?

Partly

Is the work clearly and cogently presented?

Yes

Is the topic of the essay discussed accurately in the context of the current literature?

Yes

Does the essay contribute to the cultural, historical, social understanding of the field?

Yes

Reviewer Expertise:

Higher education

I confirm that I have read this submission and believe that I have an appropriate level of expertise to confirm that it is of an acceptable scientific standard.

# Reviewer report: Reviewer response for version 1

**DOI:** 10.21956/openreseurope.18624.r40155

**Published:** 2024-06-18

**Authors:** Jasmina Berbegal-Mirabent

**Affiliations:** 1 Universitat Politecnica de Catalunya, Barcelona, Catalonia, Spain

This paper presents a promising approach by adapting the Business Model Canvas (BMC) to the context of Higher Education Institutions (HEIs). This idea is both innovative and thought-provoking, although it risks controversy if not properly explained and if the university is overly conceptualized as a business entity. I encourage the authors to support their proposal with studies that conceptualize the university as an institution with multiple objectives. Therefore, one-size-fits-all models are inappropriate. In this regard, developments such as Unicanvas can help universities customise their strategies according to their objectives, providing flexibility based on current needs and requirements. While the current arguments in the introduction lean towards this direction, some strong claims should be better supported with recent references that reinforce this perspective.

 Detailed comments:

 1) Strategic planning of HEIs: Considering that this paper is situated within the field of strategy, I believe it would be greatly enhanced by including a dedicated section that reviews existing approaches to strategic planning in Higher Education Institutions (HEIs) and their respective shortcomings. Such a section would not only strengthen the theoretical foundation of the paper but also articulate the gaps that the proposed model aims to fill.

 2) Introduction - Second paragraph: This paragraph discusses the limitations of current strategic planning models. The ideas presented are relevant but lack support from the literature. Please provide adequate references to illustrate the identified shortcomings. Referencing key studies that critique traditional strategic planning models in HEIs will strengthen the argument and provide a solid foundation for the proposed adaptation of the BMC.

 3) Introduction - Fourth paragraph: Several studies that have used or adapted the BMC in the specific context of HEIs are mentioned. However, there is no detail on how the model was adapted or the challenges encountered. Later in the second section of the paper, a review is presented, but it is limited to how the BMC was applied, not on its usefulness or the challenges faced. Providing such an analysis can help highlight which approaches were effective and which were not, allowing the new proposal to build on the experiences of existing works.

 4) Section “Origins”: The sentence “BMC is a conceptual tool that many scholars and practitioners have widely adopted” might be unnecessary in this section. It would be more effective if moved to the first mention of the BMC and its uses, providing an early context for its widespread acceptance and application.

 5) Heading “Unicanvas”: The heading “Unicanvas” on page 4 should be treated as a subheading under the section “Building Unicanvas”.

 6) Description of the building blocks: The paper does not describe the process used to develop the 10 building blocks included in the Unicanvas proposal. Who was involved in defining these blocks (e.g., panel of experts)? What process was followed? Although the paper is classified as an “essay,” some systematic approach must have been undertaken. Providing details on this process will lend credibility to the proposal and clarify the rationale behind the selected building blocks.

 7) University missions: At the beginning of the paper, the authors refer to the four missions of the university (teaching, research, technology transfer). Later, on page 6, they refer to HEIs in the context of the quintuple helix, indicating they perform teaching, research, technology transfer, and respond to society and the natural environment. Similarly, on page 6 (3rd paragraph, first column), the university’s missions are described as teaching, research, technology transfer, and a central role in regional development. The message is inconsistent and potentially misleading. While it is challenging to adhere strictly to one nomenclature or use seminal models (e.g., triple helix model), consistency is crucial. The authors should decide which references to use and maintain consistency throughout the document.

 8) Terminology consistency: Sometimes the building blocks are referred to as “categories,” which can be confusing. I recommend the authors stick to one term to avoid confusion.

Is the argument persuasive and supported by appropriate evidence?

Partly

Is the work clearly and cogently presented?

Yes

Is the topic of the essay discussed accurately in the context of the current literature?

Yes

Does the essay contribute to the cultural, historical, social understanding of the field?

Yes

Reviewer Expertise:

Higher education

I confirm that I have read this submission and believe that I have an appropriate level of expertise to confirm that it is of an acceptable scientific standard, however I have significant reservations, as outlined above.

# Response

**Published:** 2024-07-19

**Authors:** Jose M. Sanchez

**Affiliations:** Business organization, Universidad de Cadiz, Cádiz, Andalusia, Spain

Dear Jasmina, we would like to thank you for your kind comments and remarks, which were very helpful in strengthening our paper. Below we respond to the detailed suggestions that you raised:

 The abstract has been changed to reflect the reviewer's idea. On the other hand, in the introduction part, and to respond to this general comment of the reviewer, we have highlighted some trends in the context of HEIs that are making universities face different challenges and the creation of many types of value for which dynamic, flexible and innovative tools such as BMC are required. Some papers have been used to respond to this reviewer's comment. In this regard, some limitations of some classical strategic planning tools used in universities have been included. However, we have considered it appropriate not to create a new section dedicated to reviewing existing approaches to strategic planning in Higher Education Institutions, as this would distract too much attention from the central objective of the paper, which is to apply BMC in the context of HEIs. In response to your suggestion, we have included adequate references to support the shortcomings outlined above. Page 3 In the second section, and following the reviewer's recommendation, we have explained the usefulness of this tool in each of the cases in which it has been applied, highlighting that the previous works had focused on specific objectives, but none of them highlighted the versatility of the tool for designing diverse and innovative business models. Page 5 According to your suggestion, the sentence has been moved. In response to your comment, we have changed one title to another. This essay proposes the strategic tool Unicanvas after an extensive development process, which started a few years ago in a summer course at the University of Cadiz. In this course, the participants, representatives of several Spanish universities, discussed a first draft. Subsequently, using previous research cited in the manuscript as a basis, a team of experts, authors, was established. The different authors have a wide and complementary experience in the development of BMC and BSC, strategic plans for both public and private entities and universities. Several drafts have been presented in various forums to receive feedback and improvements. The present proposal is the result of this process. In the section on future lines of research, we have indicated the opportunity to design canvases with real cases and in these works explain the process of designing and filling in the blocks according to the suggestions of Mulyana et al. (2018). The reference to the concepts of the entrepreneurial university, engaged university and the triple helix model has been eliminated from the text, since, as the reviewer rightly suggests, it detracted from the consistency of the text. The role of the university in Carayannis and Campbell's model of the quintuple helix has been emphasised throughout the work, highlighting the three main functions of the university, teaching, research and transfer, and the dual social and economic mission of universities in civil society and the natural environment. Both in the introduction and the rest of the sections, the reference to this model is coherent and no different terminology has been used. Thus, the development of each of the Unicanvas blocks, we refer to the missions of the universities within this model. Pages 2 and 3. We have homogenised the terminology, using blocks instead of categories, to avoid confusion.

# Reviewer report: Reviewer response for version 1

**DOI:** 10.21956/openreseurope.18624.r40148

**Published:** 2024-06-17

**Authors:** Rafael Ventura

**Affiliations:** 1 University of Malaga, Málaga, Spain

The research is focused on a very interesting topic, addressing a challenge that universities are currently facing . Strategic management in Higher Education Institutions (HEIs) is crucial due to the dynamic and complex nature of their operational environment. HEIs face multiple challenges, including demographic shifts, technological advancements, reduced public funding, and increased competition, which necessitate a more agile and responsive strategic approach.

 The research address alternatives to the traditional static strategic planning models, often criticized for their inflexibility and top-down approach, that are becoming obsolete according to the evolving demands of HEIs.

 Effective strategic management helps universities align their resources and capabilities with their long-term objectives, ensuring they can adapt to external pressures while fulfilling their educational, research, and societal roles.

 In this vein, in a right way, is proposed an adapted version of Business Model Canvas (BMC), a dynamic tool widely used for business model innovation. It is designed to provide an intuitive, participative, and flexible framework that helps organizations conceptualize, articulate, and communicate their business model. Consequently, the paper could include references to Business Model Innovation (BMI) specifically within the university context. BMI involves the continuous adaptation and transformation of business models to create new value and competitive advantage. In the case of universities, this means developing innovative educational programs, research initiatives, and community engagement activities that respond to evolving societal needs.

 BMC’s simplicity and adaptability make it an effective tool for strategic management. It facilitates a comprehensive understanding of the university’s value creation processes and allows for continuous improvement and alignment of strategies with institutional goals and stakeholder expectations. In the context of university strategic management, it could be appropriate to specify the aspects in which the BMC is most useful and recognize the need to complement it with other tools to comprehensively address the complexity of the higher education environment. The following areas highlight the need for such complementarities: Internal and External Analysis; Performance Measurement and Evaluation; Scenario Planning; Scenario planning; and Stakeholder Analysis.

 The proposed adaptation of BMC to Unicanvas adds an 'Achievement' block, reflecting the specific non-monetary outcomes and broader impacts relevant to universities, such as reputation, societal contributions, and research outputs. The uniqueness of the university could inspire a version more aligned with the characteristics of universities. In this sense, unicanvas could include more original elements that differentiate the tool from BMC. Additionally, Unicanvas could be distinguished from other university management tools, highlighting its positioning in terms of the functionality it covers.

 Interesting work that could be improved with further adaptation of the tool to the uniqueness of the university.

Is the argument persuasive and supported by appropriate evidence?

Partly

Is the work clearly and cogently presented?

Yes

Is the topic of the essay discussed accurately in the context of the current literature?

Yes

Does the essay contribute to the cultural, historical, social understanding of the field?

Partly

Reviewer Expertise:

Business Model Innovation.

I confirm that I have read this submission and believe that I have an appropriate level of expertise to confirm that it is of an acceptable scientific standard, however I have significant reservations, as outlined above.

# Response

**Published:** 2024-07-19

**Authors:** Jose M. Sanchez

**Affiliations:** Business organization, Universidad de Cadiz, Cádiz, Andalusia, Spain

Dear Rafael, we would like to thank you for your kind comments and remarks, which were very helpful in strengthening our manuscript. Below we respond to the detailed suggestions that you raised:

 Your suggestion to review the business model literature has been followed. Specifically, some articles have been included that explain how universities should design new business models in changing environments with a multitude of challenges. Likewise, your suggestion has been taken into account by including in the objective, in the development of the blocks, and the conclusions, that unicanvas allows universities to dynamically and holistically propose and evaluate innovations to create and distribute value in response to current challenges. By enabling the participation of stakeholders to formulate, design, and validate the business concept before embarking on the more costly business plan development and implementation. Pages 2, 3 and 4 In the conclusions, it has been acknowledged that Unicanvas, despite its advantages, is not without its limitations. Once some of these limitations have been listed, its application in combination with other tools such as those mentioned above has been proposed to capture the complexity of HEIs. Pages 14 and 15 We agree with your suggestion. Unicanvas could include more original elements that differentiate the tool from BMC. Furthermore, unicanvas could distinguish itself from other university management tools by highlighting its positioning in terms of the functionality it covers. The limitations and shortcomings of traditional strategic planning models that could be overcome using Unicanvas have been included in the conclusions, specifically in the practical implications of the article. We believe that we need some empirical studies to improve this tool, adapting it more to the uniqueness of each university.

# Reviewer report: Reviewer response for version 1

**DOI:** 10.21956/openreseurope.18624.r40153

**Published:** 2024-06-14

**Authors:** Wojciech Czakon

**Affiliations:** 1 Jagiellonian University, Kraków, Poland

The study offers a much needed concept of Unicanvas, that is a development of the Business Model Canvas towards the specific needs of higher education institutions. The authors indicate that Higher Education Institutions do not have clearly defined customers, as opposed to businesses. While students constitute one group of clients, other stakeholders such as start-ups or the wider society are also relevant. Therefore, the authors suggest that the original Business Model Canvas needs to be expanded to incorporate objectives important to all stakeholders. Accordingly, they propose a 10th element to the original BMC that is outcomes at multiple levels and relative to various stakeholders. The merit of this work is conciseness and strong grounding in the business model concept. It has a descriptive, but also performative potential impact.

Is the argument persuasive and supported by appropriate evidence?

Yes

Is the work clearly and cogently presented?

Yes

Is the topic of the essay discussed accurately in the context of the current literature?

Yes

Does the essay contribute to the cultural, historical, social understanding of the field?

Yes

Reviewer Expertise:

Strategic management, interorganizational relationships, organizational resilience, antecedents-process-outcomes examination.

I confirm that I have read this submission and believe that I have an appropriate level of expertise to confirm that it is of an acceptable scientific standard.

# Reviewer report: Reviewer response for version 1

**DOI:** 10.21956/openreseurope.18624.r40150

**Published:** 2024-06-11

**Authors:** Tommaso Minola, Lucia Alejandra Rodriguez Aceves

**Affiliations:** 1 University of Bergamo (Ringgold ID: 18953), Bergamo, Lombardy, Italy

Specific comments

- **Is the topic of the essay discussed accurately in the context of the current literature?**

In general, yes, as it considers literature on strategic management, HEIs, and specifically on business models. However, it could be complemented and strengthened by adding literature on entrepreneurial universities such as:

 (Audretsch et al., 2021). 

 (Guerrero, M et al.,2019). 

 (Stolze, A. et al.,2021).

 On the other hand, regarding BM and BMC literature, the authors do an adequate job of collecting it, although the essay could benefit from including/citing more studies so as not to rely too heavily on the Gauss & Raith, 2013 article, which is used significantly throughout the document and is a conference proceeding. Here is an example from a quick search conducted on Google Scholar:

 (Talukdar, M. R. I. et al.,2021). 

 (Amit, R. et al.,2020).

  

- **Is the work clearly and cogently presented?**

Yes, the essay is presented in an orderly and clear manner, first describing what the business model canvas is and its various applications. It then describes how the co-authors built the Unicanvas and each of the 10 dimensions that integrate it. For each dimension, a definition and practical examples that HEIs can choose to integrate into their own Unicanvas are presented.

  

- **Is the argument persuasive and supported by appropriate evidence?**

The argument is persuasive and although it is supported by appropriate evidence, I believe it could be strengthened. For example, by adding an example of a hypothetical HEI, perhaps a “generic” one from a region, and showing an Unicanvas with the dimensions filled in, followed by a description of the logic for filling it in that way.

 Another possible improvement in the essay is to delve deeper into the explanation of the added dimension “achievement” and its interrelation with the “revenue stream” dimension, since although both are described, the examples offered are insufficient and somewhat confusing. This may hinder the correct application of the tool by HEI administrators. Furthermore, the “revenue streams” dimension could perhaps be renamed, considering the logic described where HEIs (at least public ones) are “Non for profit”.

 To further strengthen the theoretical implications, I would suggest citing the authors of the mentioned theories (page 8) and delving a bit into the argument of Institutional theory. Specifically, describing and explaining why such theory is valuable considering the assumption provided “the universities from the same institutional context will share some blocks”.

 Finally, also in page 8, I would suggest describing the deficiencies that have been pointed out in the BMC literature. I believe this could be valuable for HEI administrators when evaluating the use of the tool in their strategic management processes.

  

- **Does the essay contribute to the cultural, historical, social understanding of the field?**

The BMC has been used for several years now, being applied in a wide variety of organizations, including universities. However, I believe that with the examples offered, the essay contributes to the understanding of how to streamline strategic management in HEIs through the use of Unicanvas. Additionally, it promotes decision-making based on the value creation perspective, which is quite necessary in HEI management. Finally, this comprehensive perspective facilitates the identification of the various HEI stakeholders and their motivations and value exchanges.

Is the argument persuasive and supported by appropriate evidence?

Partly

Is the work clearly and cogently presented?

Yes

Is the topic of the essay discussed accurately in the context of the current literature?

Partly

Does the essay contribute to the cultural, historical, social understanding of the field?

Yes

Reviewer Expertise:

N/A

We confirm that we have read this submission and believe that we have an appropriate level of expertise to confirm that it is of an acceptable scientific standard, however we have significant reservations, as outlined above.

## References

[1] : Three-ring entrepreneurial university: in search of a new business model. *Studies in Higher Education* .2021;46(5) : 10.1080/03075079.2021.1896804 977-987 10.1080/03075079.2021.1896804

[2] : A research agenda for entrepreneurship and innovation: the role of entrepreneurial universities. *A Research Agenda for Entrepreneurship and Innovation* .2019;107-133

[3] : A meta-ethnography on HEIs’ transformation into more entrepreneurial institutions: Towards an action-framework proposition. *Industry and Higher Education* .2021;35(1) : 10.1177/0950422220922677 14-27 10.1177/0950422220922677

[4] : The Emergence of New Strategic Business Models for Heis Evidence from Bangladesh. *Academy of Entrepreneurship Journal* .2021;1-16

[5] : Business Model Innovation Strategy: Transformational Concepts and Tools for Entrepreneurial Leaders. *John Wiley & Sons.* .2020;

# Reviewer report: Reviewer response for version 1

**DOI:** 10.21956/openreseurope.18624.r40154

**Published:** 2024-05-17

**Authors:** Ursula Weisenfeld

**Affiliations:** 1 Leuphana University, Lüneburg, Germany

**Review:**

 The authors explore and adapt the Business Model Canvas (BMC), a tool to manage and change business models, in the field of Higher Education (HE): they propose Unicanvas as a tool for strategic management in HE.

 This is relevant because HE organisations (HEO) face an increasingly complex environment, owing to (amongst others) internationalization of the field, digitization in the areas of teaching and research, and digitalization in society.

 The tool can help navigating the complexity by categorizing and linking aims and activities, thereby visualizing components and interrelations of a HE organization.

 Thus, the authors’ proposal to adapt the BMC to the field of HE is relevant and useful.

 In the following I will address the authors’ characterization of the field of HE and the specifics of the tool with the aim to make some suggestions.

 The field of HE

 Asking the question ‘what is a university?’ will lead to a variety of answers and while we have seen changes in the field from mainly ‘organized anarchy with fluid participation’ (Cohen et al 1972) to more business-like organizations, the field is quite heterogeneous.

 You propose Unicanvas for “entrepreneurial universities in their quintuple helix model” (1). Because of the tool’s flexibility it can be applied to any university, thus I would not emphasize “entrepreneurial universities”, nor the “quintuple helix model” (also because you don’t explain the terms and don’t make use of this specification in the article).

 You state: “Universities are responsible for training students, generating knowledge from their research, and transferring it *to the market”* (italics added). I think the latter is too narrow – I suggest that the transfer relates more broadly to ‘society’.

 You assume the ‘marketization’ of HEOs and point to respective opportunities and the implication that universities should become more market- and customer-oriented (p. 4 and 5). However, this marketization and the question whether students are customers (rather than members) of a university are discussed critically by scholars (e.g. Gupta et al 2023, Molesworth et al 2009, Natale and Doran 2012, Parker 2014), which is worth mentioning.

 Again, because of the tool’s flexibility, it can be applied to any university (which is the beauty of the concept) and thus, I would not emphasize the special usefulness for market-oriented universities (the latter could do with the original BMC).

 The tool

 Unicanvas consists of ten building blocks: nine blocks are adapted from the BMC and a tenth building block ‘achievement’ is added.

 The idea of adapting the BMC to the field of HE is good because it might improve creating impact – I think you could be bolder in diverting from the original blocks to reflect the specifity of HE.

 You could bundle (instead of adding) and rename blocks to reflect the specific nature of HE.

 For example, students benefit from, but also contribute to the generation of knowledge.

 Unicanvas could be a useful tool for HEO to navigate complexity. I suggest to adapt the tool more closely to the nature of an HE organization:

 If a HEO is a market-oriented company, the original BMC would do. On the other end of the spectrum, if it is a public organization with the mission around knowledge creation and dissemination for the benefit of society, Unicanvas could be useful to address increased complexity and to improve impact.

**References:**

 1. Cohen M, et.al., 1972 (Ref 1)

 2. Gupta A, et.al., 2023 (Ref 2)

 3. Molesworth M, et.al., 2009 (Ref 3)

 4. Natale S, et. al., 2012 (Ref 4)

 5. Parker M, et. al., 2014 (Ref 5)

Is the argument persuasive and supported by appropriate evidence?

Partly

Is the work clearly and cogently presented?

Yes

Is the topic of the essay discussed accurately in the context of the current literature?

Yes

Does the essay contribute to the cultural, historical, social understanding of the field?

Partly

Reviewer Expertise:

innovation and change

I confirm that I have read this submission and believe that I have an appropriate level of expertise to confirm that it is of an acceptable scientific standard, however I have significant reservations, as outlined above.

## References

[1] : A Garbage Can Model of Organizational Choice. *Administrative Science Quarterly* .1972;17(1) : 10.2307/2392088 10.2307/2392088

[2] : Higher education students as consumers: a cross-country comparative analysis of students’ views. *Compare: A Journal of Comparative and International Education* .2023; 10.1080/03057925.2023.2234283 1-18 10.1080/03057925.2023.2234283

[3] : Having, being and higher education: the marketisation of the university and the transformation of the student into consumer. *Teaching in Higher Education* .2009;14(3) : 10.1080/13562510902898841 277-287 10.1080/13562510902898841

[4] : Marketization of Education: An Ethical Dilemma. *Journal of Business Ethics* .2012;105(2) : 10.1007/s10551-011-0958-y 187-196 10.1007/s10551-011-0958-y

[5] : University, Ltd: Changing a business school. *Organization* .2014;21(2) : 10.1177/1350508413502646 281-292 10.1177/1350508413502646

# Response

**Published:** 2024-07-19

**Authors:** Jose M. Sanchez

**Affiliations:** Business organization, Universidad de Cadiz, Cádiz, Andalusia, Spain

Dear Ursula, we would like to thank you for your kind comments and remarks, which were very helpful in strengthening the paper. Below we respond to the detailed suggestions that you raised:

 In response to your suggestion, with which we agree, the reference to the concepts of entrepreneurial universities has been removed from the text, since, as the reviewer suggests, these are concepts that are not developed in this manuscript, and would also limit the application of unicanvas to universities with this orientation. Precisely, one of the advantages of this tool is its versatility, which allows it to be applied to any university with any orientation, and to design tailored business models to achieve any value proposition. However, we have referred in the introduction to the quintuple helix model, where universities face their three traditional missions (teaching, research, and transfer) together with the contribution to the social and natural environment to contextualise the new ways of creating value for these institutions, the importance of developing business model innovation and to highlight that all these challenges require visual and dynamic tools such as the canvas. Pages 2 and 3 We have changed the sentence by extending the transfer process not only to the market but to the needs and demands of society as suggested by the reviewer. The consideration of the quintuple helix helps us to broaden the missions of the university. Pages 2 and 3. The articles suggested by the reviewer to reflect on the role of students in universities have been taken into account. In this sense, when analysing the "beneficiaries" block, we have reflected on the differences that Unicanvas proposes for private universities versus public universities, the former being market-oriented, and considering the student as the client, and the latter not necessarily, with the student being a resource rather than a client. After reviewing the literature, we have concluded that both public and private universities must be student-oriented, which in both cases is a key beneficiary. Page 7. We agree with the reviewer, and as pointed out in points 1 and 3, the versatility of the unicanvas tool, which can be applied to any university, has been highlighted in the paper. In this sense, we refer in the paper only to the marketisation of HEIs in the sense of broadening the beneficiaries of HEIs and to which universities can bring value. It is not necessarily market-oriented universities that should use Canvas for the design of their business models. Unicanvas incorporates four new features to BMC. First, the new name of the "customers/users" block has been changed to "beneficiaries". Second, we add “deployment” to the distribution channel block. Third, the budget is added to the cost structure block. Finally, the tenth block "achievement" has been included. However, as a future line of research, the design of the canvas in a practical way in real cases has been considered, and at that time we will reflect on the union of blocks or renaming of blocks to design business models tailored to different value propositions. It has been highlighted in the paper that unicanvas is suitable for HEIs facing complex situations and challenges. We have also highlighted that unicanvas applies to all universities, public and private as suggested by Gaus and Reith (2016). Page 10 and 11
